# Protein-Engineered Fibers For Drug Encapsulation Traceable via ^19^F Magnetic Resonance

**DOI:** 10.1021/acsanm.3c04357

**Published:** 2023-11-06

**Authors:** Dustin Britton, Jakub Legocki, Orlando Aristizabal, Orin Mishkit, Chengliang Liu, Sihan Jia, Paul Douglas Renfrew, Richard Bonneau, Youssef Z. Wadghiri, Jin Kim Montclare

**Affiliations:** † Department of Chemical and Biomolecular Engineering, New York University Tandon School of Engineering, Brooklyn, New York 11201, United States; ‡ Center for Advanced Imaging Innovation and Research (CAI2R), New York University School of Medicine, New York, New York 10016, United States; § Bernard and Irene Schwartz Center for Biomedical Imaging, Department of Radiology, New York University School of Medicine, New York, New York 10016, United States; ⊥ Center for Computational Biology, Flatiron Institute, Simons Foundation, New York, New York 10010, United States; || Center for Genomics and Systems Biology, New York University, New York, New York 10003, United States; # Courant Institute of Mathematical Sciences, Computer Science Department, New York University, New York, New York 10009, United States; ∇ Department of Chemistry, New York University, New York, New York 10012, United States; ○ Department of Biomaterials, New York University College of Dentistry, New York, New York 10010, United States

**Keywords:** protein fibers, ^19^F MRS, theranostic, drug encapsulation, imaging, biomaterial

## Abstract

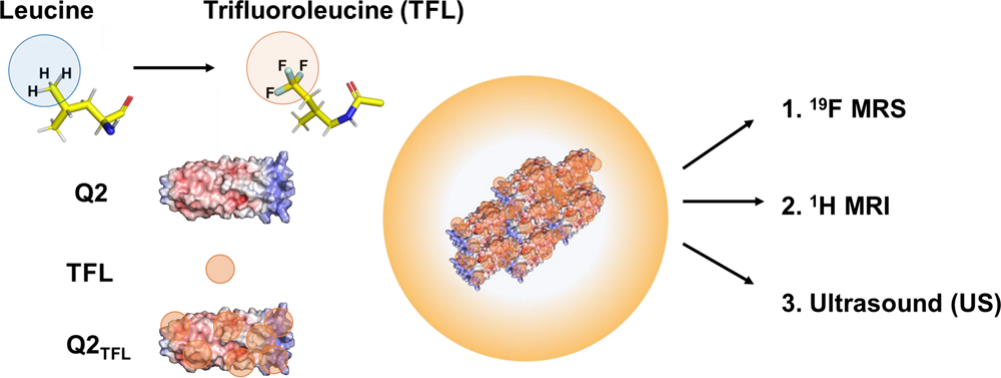

Theranostic materials research is experiencing rapid growth driven by the interest in integrating both therapeutic and diagnostic modalities. These materials offer the unique capability to not only provide treatment but also track the progression of a disease. However, to create an ideal theranostic biomaterial without compromising drug encapsulation, diagnostic imaging must be optimized for improved sensitivity and spatial localization. Herein, we create a protein-engineered fluorinated coiled-coil fiber, Q2_TFL_, capable of improved sensitivity to ^19^F magnetic resonance spectroscopy (MRS) detection. Leveraging residue-specific noncanonical amino acid incorporation of trifluoroleucine (TFL) into the coiled-coil, Q2, which self-assembles into nanofibers, we generate Q2_TFL_. We demonstrate that fluorination results in a greater increase in thermostability and ^19^F magnetic resonance detection compared to the nonfluorinated parent, Q2. Q2_TFL_ also exhibits linear ratiometric ^19^F MRS thermoresponsiveness, allowing it to act as a temperature probe. Furthermore, we explore the ability of Q2_TFL_ to encapsulate the anti-inflammatory small molecule, curcumin (CCM), and its impact on the coiled-coil structure. Q2_TFL_ also provides hyposignal contrast in ^1^H MRI, echogenic signal with high-frequency ultrasound and sensitive detection by ^19^F MRS *in vivo* illustrating fluorination of coiled-coils for supramolecular assembly and their use with ^1^H MRI, ^19^F MRS and high frequency ultrasound as multimodal theranostic agents.

## Introduction

Theranostic agents are a growing field in biomedicine that help to overcome limitations in biomaterials providing therapy and diagnosis of diseases.^1^ These materials help to monitor the development of disease after therapeutic treatment as well as provide a simultaneous diagnosis and treatment of a disease.^1^ Currently, theranostics largely focus on synthetic approaches while using inorganic materials such as quantum dots or radiolabeling to confer diagnostic properties.^1−3^ Quantum dots suffer from stability and aggregation, which greatly reduces their diagnostic sensitivity and limit their ability to effectively penetrate tissues with their signal.^4^ The practical application of radiolabeling can be challenging due to the short half-lives of radioactive isotopes, which impose logistic constraints. The resulting limited time window necessitates the use of efficient synthesis methods to ensure timely labeling. However, it also raises concerns about potential prolonged radiation exposure during the labeling process.^5^ Theranostics are also challenged by combining drug delivery techniques that possess targeting moieties with high specificity, thus reducing therapeutic efficacy and signal sensitivity.^6^ To create an ideal theranostic biomaterial, without compromising drug encapsulation, diagnostic imaging must be optimized for improved detection.^7^ One such method to improve this specificity is the incorporation of fluorine into biomaterials.^8^

Since fluorine is largely absent from organisms, yet exists in 100% natural abundance, it is useful as a contrast agent due to its specific signal in ^19^F MRS.^9^ In light of this, many ^19^F MRS materials have been developed for biomedical applications^10−14^ such as MRI cell tracking^15,16^ and tumor imaging^17,18^ as well as monitoring tumor cell hypoxia^19^ and proliferation.^20^ These agents are often synthetically derived to create fluorine-based polymers^17,21−23^ or nanoemulsions.^24−26^

With recent advancements in synthetic and chemical biology, protein engineered theranostic agents have been developed^27^ where fluorinated proteins can be produced through methods such as noncanonical amino acid (NCAA) incorporation^28,29^ or solid-phase peptide synthesis (SPPS).^30^ We have previously developed a protein-based ^19^F MRS-traceable micelle by residue-specific NCAA incorporation of trifluoroleucine (TFL) into a thermoresponsive assembled protein (TRAP), resulting in F-TRAP.^31^ Whereas previous fluorinated proteins suffer from unfavorable relaxation properties necessary to directly visualize ^19^F protein nuclei in MRI, the supramolecular micelle assembly of F-TRAP provides the opportunity for fluorine amplification due to ordering and structural constriction.^31^

Conversely, we have also shown the ability to use protein fibers for theranostic imaging by biorthogonal azide–alkyne cycloaddition of a designed coiled-coil protein with azidohomoalanine (AHA), Q_AHA_, to an alkyne-bearing iron oxide templating peptide, CMms6.^32^ The hybrid Q_AHA_-X-CMms6 bearing the templated ultrasmall superparamagnetic iron oxide (USPIO) biomaterial is capable of doxorubicin encapsulation and exhibits sensitive *T*_2_*-weighted MRI darkening in part due to the multitude of USPIOs spaced along a single protein fiber assembly.^32^ The Q_AHA_ also establishes our fibers to be capable of concentrated and sustained release.^32^ While Q highlights the benefits of using a self-assembling fiber to confine many MR-sensitive USPIOs and provides unique *T*_2_*-darkening, it suffers from the addition of several postpurification synthesis steps. In contrast, biosynthetic fluorination by NCAA incorporation of TFL is achieved in a single step.

Given the strong decrease in ^19^F *T*_2_ relaxation times as a result of F-TRAP micelle ordering and constriction as well as the evidence of the USPIO agent ordering along hybrid Q_AHA_-X-CMms6 fibers, we similarly propose that a ^19^F nuclei dense coiled-coil fiber may prove to be a sensitive ^19^F MRS theranostic agent. Fibrous biomaterials also benefit from the ability to form scaffolds for cell growth, tissue function^33,34^ as well as retain composition and localization for drug delivery.^35^ While we have previously studied the impact of TFL incorporation into Q,^36^ we have not studied the candidacy of a coiled-coil protein fiber for ^19^F MRS.

Herein, we develop a protein-based fluorinated self-assembling fiber, Q2_TFL_ as a theranostic agent capable of ^19^F MRS. We demonstrate that Q2_TFL_ has increased sensitivity for ^19^F MRS, and increased thermostability compared to previous constructs, and can encapsulate the hydrophobic small molecule, curcumin (CCM), which provides further stabilization. Furthermore, we show that Q2_TFL_ may be used *in vivo* as a visible fiber assembly via ^1^H MRI and high-frequency ultrasound as well as a sensitive biomaterial using ^19^F MRS. Interestingly, we show that Q2_TFL_ possesses a ratiometric ^19^F MRS signal proportional to its protein structure and environmental temperature indicating its potential as a multifunctional *in vivo* probe.

## Materials and Methods

### Materials

Electrically competent LAM1000 *E. coli* cells^37^ were gifted from David Tirrell at California Institute of Technology. Bacto-tryptone, sodium chloride (NaCl), yeast extract, tryptic soy agar, ampicillin sodium salt, sodium phosphate dibasic anhydrous (Na_2_HPO_4_), sodium hydroxide (NaOH), dextrose monohydrate (d-glucose), magnesium sulfate (MgSO_4_), calcium chloride (CaCl_2_), manganese chloride tetrahydrate (MnCl_2_·4H_2_O), cobaltous chloride hexahydrate (CoCl_2_·6H_2_O), isopropyl β-_D_-1-thiogalactopyranoside (IPTG), Pierce bicinchoninic acid (BCA) assay kit, Pierce snakeskin dialysis tubing 3.5 K molecular weight cutoff (MWCO), sodium dodecyl sulfate (SDS), Nunc ninety-six well plates, BD Clay Adams glass microscopy slides, Pierce C18 tips, and 5,5,5-trifluoroleucine were acquired from Thermo Fisher Scientific. The 20 naturally occurring amino acids, dimethyl sulfoxide (DMSO), nickel(III) chloride hexahydrate (NiCl_2_·6H_2_O), sodium molybdate dihydrate (Na_2_MoO_4_·2H_2_O), iron(III) chloride (FeCl_3_), iron(II) chloride tetrahydrate (FeCl_2_·4H_2_O), thiamine hydrochloride (vitamin B), thioflavin T (ThT), curcumin (CCM), trifluoroacetic acid (TFA), ProteoMass peptide and protein MALDI-MS calibration kit containing sinnapinic acid, D_2_O, and copper(II) sulfate pentahydrate (CuSO_4_·5H_2_O) were purchased from Sigma-Aldrich. Hydrochloric acid (HCl), Coomassie Brilliant Blue G-250 were purchased from VWR. HiTrap FF 5 mL columns for protein purification were purchased from Cytiva Life Sciences. Macrosep and Microsep Advance Centrifugal Devices 3K MWCO and 0.2 μm syringe filters were purchased from PALL. Acrylamide/bis solution (30%) 29:1, and natural polypeptide sodium dodecyl sulfate–polyacrylamide gel electrophoresis (SDS-PAGE) standard were purchased from Bio-Rad. Copper(II) chloride anhydrous (CuCl_2_), sodium selenite (Na_2_SeO_3_), and imidazole were purchased from Acros Organics. Formvar/carbon-coated copper grids (FCF400-Cu) and 1% uranyl acetate for transmission electron microscopy were purchased from Electron Microscopy Sciences. Borosilicate glass capillaries (0.2 × 2 × 75 mm) were purchased from VitroCom.

### Expression and Purification

Q2_TFL_ and Q_TFL_ proteins were expressed as described previously.^36^ While and pQE30/Q^38^ was used from our prior studies, pQE60/Q2^39^ plasmid was cloned and purchased from Genscript and Integrated DNA Technologies, respectively. Q and Q2 were expressed in leucine auxotrophic LAM1000 *E. coli* cells in supplemented M9 minimal media. Prior to induction, expression media was allowed to grow to an optical density at 600 nm (OD_600_) of 0.8–1.0 before pelleting at 5000 × g at 4 °C for 30 min in an Avanti J-25 centrifuge (Beckman Coulter). Cells were washed a total of three times by resuspending in 0.9% NaCl previously stored at 4 °C overnight, centrifuging to repellet the cells in between washes. Following the final wash and centrifugation cycle, the cell pellet was resuspended in M9 media supplemented instead with 19 amino acids (minus leucine) and containing all other media chemicals. The expression culture was then incubated for 15 min at 37 °C and 350 rpm allowing for recovery while starving of leucine before addition of 555 μg/mL of TFL and 200 μg/mL of IPTG to induce expression. After incubation at 37 °C and 350 rpm for 3 h, cells were harvested by centrifugation at 5000 × g at 4 °C for 30 min in an Avanti J-25 centrifuge (Beckman Coulter) and stored at −20 °C until purification. 12% SDS-PAGE was used to confirm expression of Q_TFL_ and Q2_TFL_. Protein was purified using affinity chromatography on a cobalt-charged HiTrap IMAC FF 5 mL column with Buffer A (50 mM Tris-HCl, 500 mM NaCl, pH 8.0). Protein was eluted using a gradient of Buffer B (50 mM Tris-HCl, 500 mM NaCl, 500 mM imidazole, pH 8.0) possessing an imidazole concentration range from 10–500 mM. Pure fractions were then dialyzed in six consecutive 5 L volumes of Buffer A and concentrated to approximately 2 mM using 3 kDa Macrosep centrifugal filters (Pall). Protein purity was confirmed by 12% SDS-PAGE and concentration determined by BCA assay.

### Assessment of Trifluoroleucine Incorporation

Trifluoroleucine (TFL) was assessed by matrix-assisted laser desorption/ionization- time-of-flight mass spectrometry (MALDI-TOF MS) using a Bruker UltrafleXtreme MALDI-TOF/TOF. Protein was diluted 1:50 in water before being mixed in equal parts diluted sample to sinnapinic acid matrix. Protein sample was spotted onto a Bruker MTP 384 steel target plate and vacuum-dried in a desiccator. Using the same protocol, Sigma-Aldrich peptide standards were also spotted onto the target plate. The spectra were then deconvoluted to Gaussian functions in PeakFit software to its maximum goodness of fit by *R*^2^ value using one peak to represent full incorporation, and ≥1 peak to represent masses less than full incorporation. The relative percent area of the incorporated Gaussian peak was used to determine the incorporation based on *n* number of peaks deconvoluted and if the Gaussian fit peak of the expected TFL peak was less than the expected *m*/*z*, the % difference was incorporated into the assessment (eq 1).
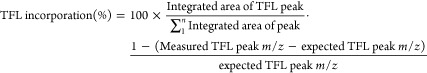
1

### Circular Dichroism Spectroscopy

The secondary structures of Q2_TFL_ and Q_TFL_ were assessed using a Jasco J-815 circular dischroism (CD) spectrometer with a PTC-423S single position Peltier temperature control system. Wavelength scans were performed from 195 to 250 at 1 nm step sizes by diluting the protein into water (at approximately 10 μM) in order to minimize the effects of sodium chloride. Temperature scans were performed from 20 to 85 °C in water and in phosphate buffer (50 mM Na_2_HPO_4_, pH 8.0) and in phosphate buffer in the presence of saturated CCM (as determined by binding data) at 1 °C/min as done previously.^40^ The mean residue ellipticity (MRE) was calculated as described in previous studies.^41^

### Attenuated Total Reflectance-Fourier Transform Infrared Spectroscopy

Secondary structure of Q2_TFL_ and Q_TFL_ protein was confirmed using attenuated total reflectance-Fourier transform infrared (ATR-FTIR) spectroscopy with a Nicolet 6700 Fourier Transform Infrared Spectrometer equipped with a diamond ATR accessory and a mercury cadmium telluride (MCT)-A detector. Spectra were collected for 5 μL of 1 mM protein from 4000 to 400 cm^–1^ with 4.0 cm^–1^ increments. Sample spectra were normalized using buffer background and analyzed from 1700 to 1600 cm^–1^ corresponding to the amide I region. Peaks were deconvoluted using Gaussian functions in PeakFit software until the goodness of fit reached r^2^ ≥ 0.99.^42,43^

### Curcumin Binding

CCM was bound to Q2_TFL_ as described previously.^40^ Briefly, increasing ratios of CCM:Q2_TFL_ were made at final volumes of 1 mL with final concentrations of Q2_TFL_ at 15 μM and a final concentration of 1% v/v DMSO. Samples were loaded onto a 96-well black plate and excited at 420 nm, and emission was read at 520 nm using a BioTek Synergy H1 microplate reader at room temperature (RT). Normalized relative fluorescence intensities were calculated and analyzed in Graphpad Prism (GraphPad Software). Binding affinity was calculated using the specific binding kinetics equation.

### Transmission Electron Microscopy

Transmission electron microscopy (TEM) images were taken with an FEI Talos L120C transmission electron microscope. Samples were diluted to 50 μM and 3 μL was spotted on Formvar/carbon-coated copper grids followed by a 5 μL wash with water and 3 μL staining with 1% v/v uranyl acetate solution each with incubation times of 1 min. Between steps, filter paper was used to wick the grids. Following imaging, fibrils were sized in ImageJ software (Version 1.52q).^44^

### Confocal Microscopy

Q2_TFL_ was diluted to 50 μM and saturated with 40 μM curcumin (solubilized in DMSO) as determined by the binding affinity in drug-binding experiments. The final concentration of samples for confocal microscopy possessed 1% v/v DMSO. Five μL of sample was deposited onto a microslide and covered with a 22 × 22 mm #1 microscope cover glass. Images were taken with a Leica TCS SP8 X laser confocal microscope using a dry 10x objective at RT. Samples were excited at 460 nm and images were taken with a 470–550 nm detection window.

### ^19^F Nuclear Magnetic Resonance

^19^F detection was studied using a Bruker AVIII-500 (11.7 T) nuclear magnetic resonance (NMR) instrument equipped with a broadband BB(F)O CryoProbe. One-pulse sequence was used to acquire the ^19^F signal with a spectral width 113,636.4 Hz corresponding to 241.5 ppm, 0.577 s acquisition time, and 256 scans. 1D ^19^F NMR spectra of Q_TFL_ and Q2_TFL_ in 10% v/v D_2_O were collected in the approximate range of 0.25–2.0 mM based on concentrations measured by BCA assay following dilution in 10% v/v D_2_O spiked buffer (50 mM Tris, 500 mM HCl, pH = 8.0). 90% TFA/10% D_2_O was acquired with the same sequence for comparison. Topspin 3.2 software was used to visualize spectra and quantify the signal-to-noise ratio (SNR) using the Bruker SINO command by calculating the ratio of the peak amplitude (signal) to the standard deviation of the noise level in the spectrum. To facilitate a comparison of SNR signals between Q2_TFL_ and F-TRAP,^31^ we estimated the gain in SNR for F-TRAP when measured at 9.4 T and translated into an 11.7 T magnetic field strength. This estimation was made under the assumption of identical experimental conditions and negligible differences in relaxation times, utilizing a simplified version of the relationship between SNR and magnetic field strength, *B*_o_, as .^45,46^

*T*_1_ and *T*_2_ relaxation times of the fluorine nuclei in Q2_TFL_ were examined using the inversion recovery and Carr–Purcell–Meiboom–Gill pulse sequences, respectively. The *T*_1_ measurement was performed with variable inversion times (TI) of 0.001, 0.05, 0.1, 0.25, 0.8, 1.5, 3.0, and 5.0 s and a 4 s repetition time (TR), averaged over 200 scans. The *T*_2_ measurement was conducted using variable echo times (TE) of 0.002, 0.02, 0.05, 0.1, 0.2, 0.4, 0.6, 0.8, 1, 1.4, 1.6, 1.8, 2.5, 5, 10, and 20 ms with a 4 s TR, averaged over 512 scans. *T*_1_ and *T*_2_ relaxation times were calculated based on a monoexponential fitting analysis using Graphpad Prism software.

### Phantom and *In Vivo* Magnetic Resonance Imaging

Magnetic resonance imaging (MRI) and spectroscopy (MRS) were performed on a Biospec 70/30 micro-MRI system (Bruker – Billerica MA, USA) equipped with zero helium boil-off 300 mm horizontal bore 7-T (7-T) superconducting magnet (300 MHz) based on ultrashield refrigerated magnet technology (USR). The magnet is interfaced to an actively shielded gradient coil insert (Bruker BGA-12S-HP; OD = 198 mm, ID = 114 mm, 660 mT/m gradient strength, 130 μs rise time) and powered by a high-performance gradient amplifier (IECO, Helsinki – Finland) operating at 300*A*/500 V. This installation is controlled by an Avance-3HD console operated under Paravision 6.1 and TopSpin 3.1. The MR imaging and spectroscopy setup utilized in this study involved the in-house design of two distinct radiofrequency (rf) resonators for scanning a mouse body. The first coil was a volume transmit-receive linear birdcage rf coil with 16 rungs, possessing an outer diameter (OD) of 72 mm, an inner diameter (ID) of 42 mm, and a length (L) of 64 mm (Figure S1a). This rf coil was tuned to 300.16 MHz, corresponding to the ^1^H proton Larmor frequency. It served the purpose of transmitting and receiving signals during the imaging process, providing radio frequency coverage for the mouse body. A rectangular flexible rf coil was also designed to enable specific detection of the fluorine (^19^F) nuclei (282 MHz) at 7 T. This flexible surface coil was fabricated by attaching adhesive flat copper tape circuitry affixed to a sheet transparency film. The coil had dimensions of *L* = 10 mm and a width (*W*) of 30 mm (Figure S1b). The coil incorporated four distributed fixed ceramic capacitors (Kyocera Co Ltd., Kyoto, JP), which facilitated electrically balanced tuning to a frequency near 282 MHz, corresponding to the ^19^F Larmor frequency.

The flexible rf coil was skillfully wrapped into the inner part of the cylindrical birdcage rf coil and positioned to optimize inductive coupling and radiofrequency (rf) coverage (Figure S1c). This configuration enabled the achievement of dual resonance for both ^1^H and ^19^F nuclei by utilizing the single port of the volume birdcage coil. The single port was connected to a tune/match box, which served as an interface between the volume coil and the spectrometer and also enabled the fine-tuning readjustment of either the proton or fluorine resonances (Figure S1d). The utilization of this single port dual-resonance setup via mutual inductive coupling facilitated the acquisition of imaging and spectroscopy data for both proton (^1^H) and fluorine (^19^F) signals, allowing for comprehensive analysis and investigation in the study. A set of ^19^F magnetic resonance spectra were acquired for the calibration and characterization of the custom-designed RF coil setup and overall sensitivity. The acquisition parameters included a TR= 5 s to enable full magnetization recovery, a number of averages (*N*_av_) = 1, and 4096 sample points for the acquisition with a spectral width (SW) = 85.227 kHz corresponding to 301.6 ppm resulting in spectral resolution of 21 Hz/pt.

A set of water phantoms doped with copper sulfate (CuSO_4_: 1 g/L, 4.01 M) and NMR tubes filled individually with 100 μL of 100% water, 13 mM trifluoroacetic acid (TFA, 100%) and 1 mM Q2_TFL_ were used for characterizing the ^1^H/^19^F rf coil set coverage, sensitivity, and performance. After conducting rf power and shim calibrations using the ^1^H signal, serial dilutions of TFA NMR tubes were utilized as a reference to evaluate the limit of detection (LOD) for the ^19^F signal in our experimental setup. The LOD was established by determining the concentration that achieved a SNR above 3 standard deviations of the noise floor.^47^ The ^19^F signal optimization of Q2_TFL_ was subsequently carried out. To achieve a constant scan time of 4 min, TR was incrementally increased from 50 to 1000 ms by adjusting the number of averages. The objective of this optimization was to determine the best combination of TR and Nav to acquire Q2_TFL_ spectra with maximum sensitivity under 4 min. Additionally, the same optimization process was repeated at a reduced acquisition time of 1 min to evaluate the impact of improved temporal resolution on SNR. The SNR values were calculated using the “sino” command in Bruker Topspin software. Specifically, the ^19^F signal interval was defined between −50 ppm and −100 ppm, while the background noise region was selected within the 0 ppm −50 ppm chemical shift range. The spectra were set to a line broadening (LB) value of 30 for display purposes only. By following this experimental protocol, the calibration of the rf power, shim adjustments, and optimization of TR were achieved, ensuring accurate NMR spectral acquisition and analysis for the Q2_TFL_ samples. Specifically, an optimization between length of the scan time and TR was found prior to *in vivo* experiments to maximize SNR of Q2_TFL_ in ^19^F MRS. Here, scan times were used at 1 min for dynamic studies and 4 min for static studies, where the number of averages was varied at different TR.

In our *in vivo*^1^H MRI/^19^F MRS mouse experiments, we opted to utilize isoflurane as the preferred anesthetic agent. Isoflurane stands as the predominant choice in small animal studies due to its advantages, including ease of administration, rapid onset, and swift offset of action. These attributes collectively contribute to a streamlined and highly predictable experimental workflow.

Our decision to employ isoflurane was influenced by the specific focus of our study, which involves the measurement of fluorine signals. Concerns regarding the potential interference from isoflurane-derived fluorine background signals prompted careful consideration. It is imperative to acknowledge that isoflurane may introduce unwanted signals that could overlap with other peaks, such as those arising from Q2_TFL_.

By contrast, for previous investigations involving the F-TRAP biomaterial,^31^ we chose to utilize ketamine-xylazine (KX) – a regulated and controlled injectable anesthetic devoid of any ^19^F background signal. However, the use of KX anesthesia carries its own set of limitations, including an irreversible and relatively extended duration of action, which can result in prolonged recovery times. These limitations have the potential to significantly impact the experimental timeline and data collection significantly.

Therefore, our preference for isoflurane over KX anesthesia was grounded in the pursuit of a smoother and more predictable experimental workflow. Isoflurane provides greater control and reversibility in terms of adjusting the depth of anesthesia during the experimental sessions. This level of control is crucial not only during the initial testing and characterization phases, where precise timing is often challenging to predict, but also in the context of long-term disease studies, where maintaining a stable physiological state remains paramount.

Following isoflurane induction, the lower body of the mouse was centered within the rf coil and positioned at the isocenter of the magnet to ensure comprehensive anatomical coverage, with the knee closely fitting the rectangular surface coil. To provide anatomical context, a ^1^H MRI scan was performed using a 3D gradient echo (3D-GE) Flash sequence. The scan parameters were set as follows: TR= 40 ms, echo time (TE) = 2.1 ms, flip angle (FA) = 30°, matrix size (Mx) = 256 × 128 × 128, field of view (FOV) = 51.2 × 25.6 × 25.6 mm, *N*_AV_ = 2. The acquisition time for this scan was less than 22 min, resulting in an isotropic image resolution of 200 μm. The primary objective of this scan was to accurately visualize the intra-articular location of Q2_TFL_ injected at 1 mM (50 μL) within the mouse.

For ^19^F scans, the rf coil resonance was readjusted to fine-tune/match the 282 MHz Larmor frequency and to perform phantom MRI settings utilized as a reference, with an acquisition time of 10 min (TR = 100 ms, *N*_AV_ = 6000). This setup ensured consistent imaging conditions for the ^19^F scans, enabling an accurate comparison and analysis of the Q2_TFL_ biomaterial.

To assess and compare the ^19^F MRS sensitivity of the current Q2_TFL_ biomaterial with that of a previously studied TFL-incorporated construct called F-TRAP,^31^ we adjusted the scan time to 6 min. 40 s (TR= 100 ms, *N*_AV_ = 4,000). This modification allowed us to evaluate the overall performance of both the experimental rf coil setup and biomaterials. Importantly, these adjustments were implemented while maintaining the MRI settings optimized for Q2_TFL_ and utilizing the same coil setup employed throughout this study for both *in vitro* and *in vivo*. Consequently, the following parameters were employed: 4096 points and SW = 85.227 kHz, resulting in a spectral resolution of 21 Hz per data point.

### Ultrasound Guided Injection

The image-guided intra-articular injection of the Q2_TFL_ was performed using a Vevo 3100 high-frequency ultrasound (US) system (Visualsonics/Fujifilm, Toronto ON, CA). The system was equipped with an adjustable rail system designed for small animal handling, precise positioning, and optimization. This setup allowed for noninvasive *in vivo* imaging under accurate physiological conditions, which included a temperature-controlled heated stage, gas anesthesia, and a syringe injection system for simultaneous compound administration.

A 50 MHz high-frequency US transducer (MX700 D) was utilized, providing an axial resolution of 30 μm and enabling real-time imaging at a rate of up to 300 frames per second. To ensure optimal imaging conditions, mice were positioned in a dorsal recumbent posture on the US heated stage. The hind limbs were flexed and externally rotated approximately 45° while a surgical tape was applied to immobilize the limbs and facilitate access to the knee joint.

Prior to the injection, a sterile US gel was applied over the joint area to enhance visualization and guidance during the injection process. The US transducer was positioned parallel to the femur, allowing for clear visualization of the patellar ligament, which appeared as a dark band in the ultrasound image.

For the injection itself, a needle was carefully inserted laterally into the patellar tendon within the joint capsule. The Q2_TFL_ (1 mM, 50 μL) solution was slowly infused through the needle, while the intra-articular release was continuously monitored using ultrasound imaging.

By employing this technique, the image-guided intra-articular injection of the Q_2TFL_ was successfully performed, ensuring reproducible targeting and delivery of the compound within the joint space while allowing for real-time monitoring of the injection process.

### Statistical Analysis

GraphPad Prism (GraphPad Software) was employed for statistical analysis using a student’s *t*-test.

## Results and Discussion

### Rationale and Protein Synthesis

**Figure 1:**
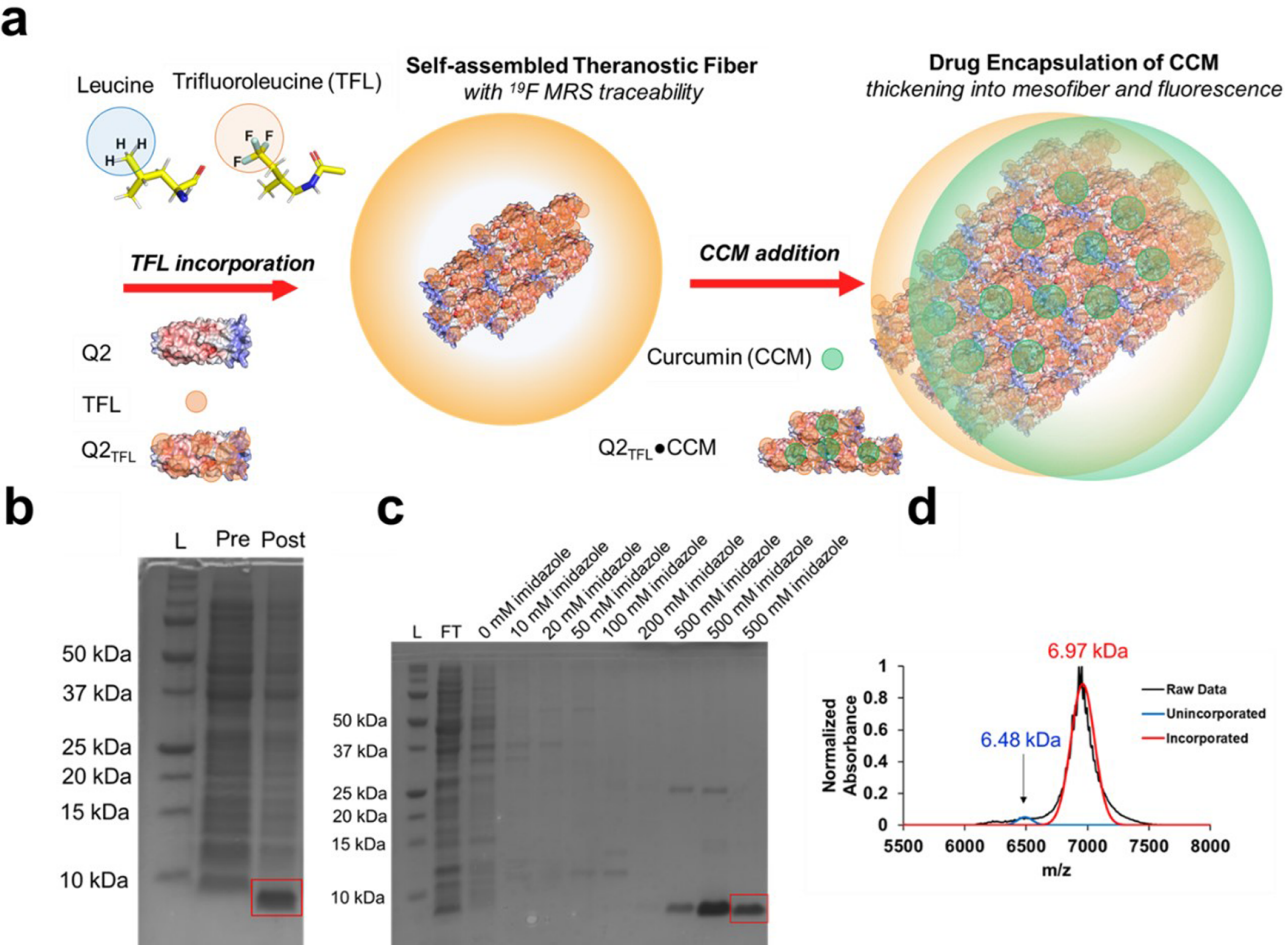
(a) Scheme of TFL incorporation and CCM encapsulation to generate Q2_TFL_ and Q2_TFL_•CCM (b) of Q2_TFL_ protein (6.97 kDa) after expression. L: Ladder, Pre: Preinduction with IPTG, Post: Postinduction with IPTG. (c) Q2_TFL_ protein after purification. L: Ladder, FT: Flow-through, following are increasing concentrations of imidazole. (d) Representative MALDI-TOF spectra showing incorporation of TFL into Q2 by the size increase to 6.97 kDa.

Q2_TFL_ was designed for greater thermostability possessing 9 leucines, compared to 7 in Q_TFL_, which was confirmed by a lower Rosetta Score^39,40^ with the aim of creating a fluorinated fiber capable of curcumin (CCM) encapsulation (Figure 1a). Q2_TFL_ was generated by residue-specific noncanonical amino acid incorporation of trifluoroleucine (TFL) using leucine auxotrophic LAM1000 *E. coli* cells.^37,48^ Protein expression (Figure 1b) and purification (Figure 1c) were assessed by 12% SDS-PAGE gels showing protein bands at a molecular weight of 6.97 kDa for Q2_TFL_. Percent of TFL incorporation was assessed using MALDI-TOF based on the molecular weight of Q2 (6.48 kDa) (Table S1). Q2_TFL_ showed an expected increase in molecular weight upon incorporation of TFL of 0.49 kDa based on the difference in molecular weight of TFL (185.14 Da) and leucine (131.17 Da) and the number of leucines. Using best-fit Gaussian peaks based on the expected molecular weight of incorporated and unincorporated proteins, Q2_TFL_ was determined to have an average incorporation of 95.0 ± 2.3% (Figure 1d, Figure S4, and Table S2) with this value near the expected range based on previous incorporation levels for TFL in coiled-coils from previous studies, which ranged from 90.7% – 95.1%.^36^ As a control, Q_TFL_ was biosynthesized, purified and confirmed for TFL incorporation as previously described (Figures S2 and S5 and Tables S1 and S3).

### Fluorinated Coiled-Coil Structure

**Figure 2:**
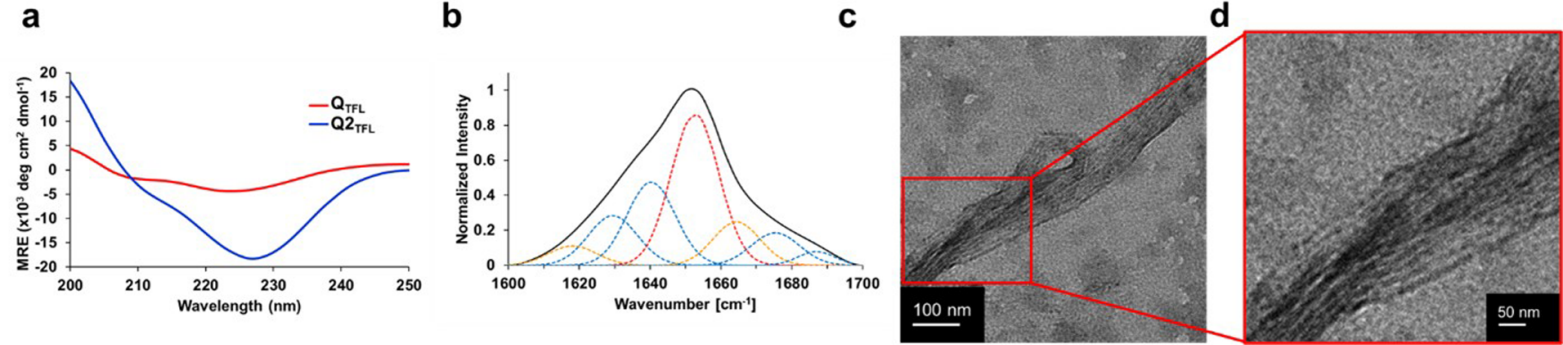
(a) CD wavelength scan of Q_TFL_ and Q2_TFL_ in water performed at 20 °C from 195 to 250 nm. Spectra are the average of three independent trials. (b) Representative ATR-FTIR spectra of Q2_TFL_. Overall spectra by deconvolution are in black and individual peak deconvolutions are in dotted red lines (α-helix), blue lines (β-sheet), and orange lines (random coil/turns). (c) Transmission electron micrograph of the Q2_TFL_ protein. (d) Higher resolution micrograph of Q2_TFL_ protein highlighting striations composing the fiber.

The secondary structure of Q2_TFL_ was assessed by using CD spectroscopy. Q2_TFL_ exhibited a characteristic α-helical spectrum with a double minimum of −100 deg cm^2^ dmol^–1^ and −15 000 deg cm^2^ dmol^–1^ at 208 and 222 nm, respectively (Figure 2a, Table S4). Additionally, Q2_TFL_ possessed a 222/208 ratio of 150. The large magnitude of the 222/208 ratio suggests that α-helices were found in proximity of other α-helices reflecting the coiled-coil and fibrous nature of Q2_TFL_.^49−51^ To further explore the impact of the higher TFL content in Q2_TFL_, we compared the data with the previously fluorinated fiber, Q_TFL_.^36^ The parent Q_TFL_ exhibited a double minimum of −500 deg cm^2^ dmol^–1^ and −4,300 deg·cm^2^·dmol^–1^ at 208 and 222 nm, respectively and a 222/208 ratio of 8.6 (Figure 2a, Table S4). Strikingly, Q2_TFL_ demonstrated a much stronger coiled-coil structure and α-helical characteristic minimum at 222 nm, in agreement with previous studies of fluorination on coiled-coil structure.^37,52−54^

In addition, the Q2_TFL_ secondary structure in its native buffer conditions was assessed using ATR-FTIR of the samples at 2 mM (Figure 2b). In agreement with CD results, Q2_TFL_ revealed a helical content of 38.4% after deconvolution (Table S5), which was 13.6% higher than the parent Q_TFL_ (Figure S5, Table S5), indicating the positive effect of additional TFLs on the coiled-coil structure.

### Supramolecular Assembly and Microstructure

Given the nature of the Q proteins to undergo supramolecular assembly at low temperatures,^39^ Q2_TFL_ was incubated at 4 °C after concentration to 2 mM in 50 mM Tris, 500 mM NaCl, pH 8.0 buffer. Q2_TFL_ underwent supramolecular assembly into nanofibers. Lower resolution micrographs showed Q2_TFL_ fiber morphology to appear similar to those found for Q_TFL_^36^ containing large diameter and sheet-like structures (Figure 2c, Figure S6). Higher resolution micrograph images revealed a fibrous structure composed of striations measuring 3.6 ± 0.8 nm in size (*n* = 20) (Figure 2d, Figure S6), approximately the diameter of a single coiled-coil domain and in agreement with the 3.5 ± 0.5 nm protofibril diameters measured in Q previously and suggesting a similar end-to-end stacking mechanism.^55^

Overall, fiber assemblies are measured to be 215.8 ± 38.6 nm (*n* = 20) in size by TEM (Figure 2c, S6). The large standard error is explained by the presence of large fiber aggregates as large as 840 nm in diameter. As a result, we view the median diameter, 181.7 nm, as a better representation of a typical fiber diameter. Whereas we have previously strongly associated nanofibril diameter size with the electrostatic potential of protofibril termini,^40,55^ the increase in diameter of Q2_TFL_ fibrils suggests the size can also be modulated by hydrophobicity, namely by fluorinating or modifying the number of hydrophobic residues lining the coiled-coil pore. To this extent, this agrees with phenomena associated with fiber thickening upon the introduction of hydrophobic small molecule curcumin (CCM) in our fibers. Strong interaction of CCM in the hydrophobic pore and in between fibers causes hydrophobic residues to be further buried and increases the exposure of nonpolar residue groups and thus increases protein surface activity.^55−57^ We similarly associate the introduction of hydrophobic residues with increased hydrophobic residue packing and surface activity, which in turn increases protofibril interaction, resulting in a population of larger fiber diameters.

**Figure 3:**
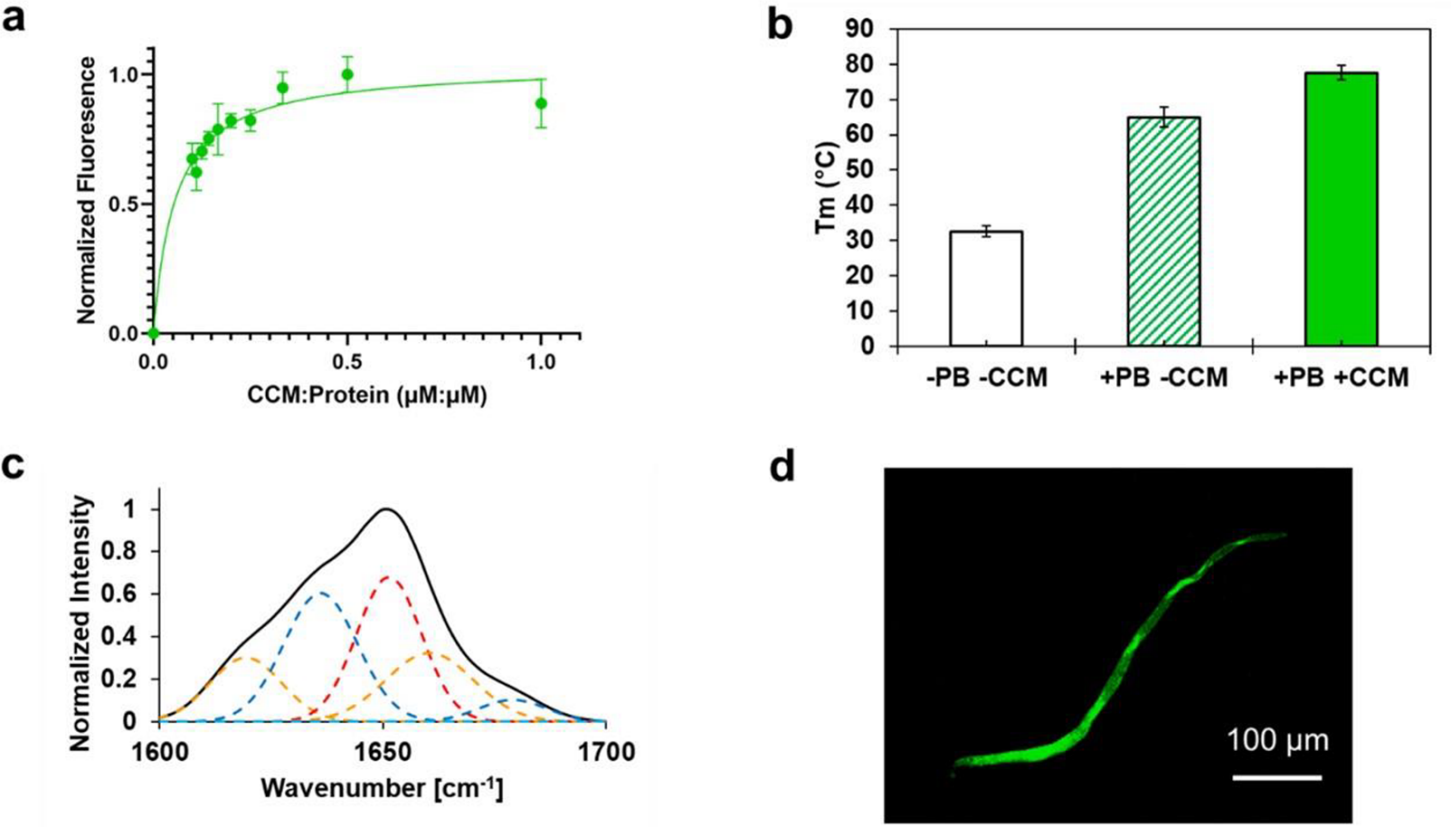
(a) Spectroscopic fluorescence of Q2_TFL_ at different curcumin:protein molar ratios. Fluorescence was measured by excitation at 450 nm and emission at 520 nm. Error bars represent the standard deviation and are the result of three independent trials. (b) Melting temperature of Q2_TFL_ in the presence of phosphate buffer (PB) with and without CCM. Melting temperature is measured by CD and error bars are the result of three independent trials. (c) Representative ATR-FTIR spectra of Q2_TFL_•CCM. Overall spectra by deconvolution are shown in black and individual peak deconvolutions in dotted red lines (α-helix), blue lines (β-sheet), and orange lines (random coil/turns). (d) Fluorescent confocal micrograph of Q2_TFL_•CCM showing fiber thickening to the mesoscale.

Q2_TFL_ thermostability was measured by CD temperature scans from 20 to 85 °C (Figure S7). In only water, in the absence of salts or buffers, Q2_TFL_ exhibited a melting temperature of 32.6 ± 1.6 °C (Figure 3a). However, under physiologically relevant buffer conditions such as the phosphate buffer used in this study, Q2_TFL_ possessed a melting temperature of 65.0 ± 2.9 °C. This range spans physiological temperature where Q2_TFL_ meets the criteria of an ionic strength-responsive protein biomaterial for controlled drug release.^58^ In previous studies,^36^ it was observed that Q_TFL_ exhibited an increase in melting temperature, rising from 39 to 52 °C. This substantial enhancement in thermostability aligns with previous research indicating that fluorinated coiled-coils tend to improve stability.^37,52−54^ Notably, a higher content of TFL resulted in a more significant increase in stability, highlighting the relationship between the TFL concentration and improved stability.

### Curcumin Binding

Coiled-coil proteins have traditionally been studied for their hydrophobic small molecule-binding ability due to the presence of a hydrophobic pore.^32,36,40,59^ In particular, curcumin (CCM)^36,40^ has been used as a model candidate drug due to its therapeutic use as an antiproliferative, antibacterial, and anti-inflammatory agent.^60−62^ We assess the ability of Q2_TFL_ to bind CCM (Figure 3b) and the impact of encapsulation on Q2_TFL_ structure and stability, where Q2_TFL_ exhibits a K_d_ of 0.06 μM/μM [CCM:protein] (0.03–0.09 μM/μM @ 95% CI), which translates to an 8:1 ratio of monomer to CCM, a significant increase compared to Q2. We use 2*K*_d_ or a ratio of 0.12 to mark saturation of CCM binding and where a negligible increase in fluorescence is seen.^40^ Moreover, CCM-bound Q2_TFL_ (Q2_TFL_·CCM) exhibits a 12.6 °C increase in melting temperature to 77.6 ± 2.0 °C (Figure 3b) via CD, which is consistent with previously reported increases upon CCM binding.^40^

ATR-FTIR was used to decipher the secondary structure of Q2_TFL_ (Figure 2b) and compared to Q2_TFL_·CCM (Figure 3c). After deconvolution of the spectra, Q2_TFL_ exhibited 42.4 ± 8.6% α-helical content, 38.4 ± 14.0% β-sheet content, and 19.2 ± 9.7% random coil content (Table S5). Upon binding to CCM, noted by broadening of the ATR-FTIR spectra, Q2_TFL_·CCM possesses 30.8 ± 6.9% α-helical content, 33.0 ± 13.7% β-sheet content, 33.3 ± 8.3% random coil content (Table S5) exhibiting a 14.1% loss in ordered structure–a behavior consistent with previous fiber·CCM binding studies.^40^

We have previously established a linear model^40^ correlating the increase in thermostability upon binding CCM, and the loss of ordered structure as measured by ATR-FTIR.^40^ Based on this model, a 14.5% loss of structure is predicted, which translates to an error of just 0.4% from our measured structure loss of 14.1%, within the root mean squared error (RMSE) of the model,^40^ which is calculated here to be 0.9%. These results both validate the linear model and strengthen our conclusion that Q2_TFL_ possesses similar binding behavior to nonfluorinated fibers previously studied. While CCM-binding imposes a negative impact on the ordered structure of the coiled-coil, a loss of secondary structure measured by ATR-FTIR has been associated with a positive interaction of CCM in the hydrophobic pore, which helps stabilize the protein and increase thermostability.^40^ Thus, the fluorination of Q2_TFL_ results in a strengthened interaction with CCM.

Binding of CCM causes fiber thickening of Q2_TFL_ (Figure 3d, Figure S8), consistent with our recent analysis of supramolecular coiled-coil fibers.^40^ Fiber-thickening by CCM has been established for collagen activity^56,57^ as well as all coiled-coil fibers designed in our lab so far^40,55^ and is explained by increased solvation of polar groups and burying of the hydrophobic residues leading to increased surface activity.^56,57^ The average fiber diameter of 10.8 ± 5.4 μm is similar to the median fiber diameter predicted by our recently established relationship between the nanofiber and CCM-thickened fiber diameters. While this relationship was assessed for nonfluorinated CCM fibers, the predicted fiber diameter is 12.9 μm based on a 181.7 nm Q2_TFL_ fiber diameter translating to an error of 2.1 μm, just outside our model’s root mean squared error (RMSE) of 0.8 μm (Figure S9). These results suggest that Q2_TFL_ supramolecular fiber assembly upon CCM-binding remains similar to our previous nonfluorinated constructs.

### ^19^F Nuclear Magnetic Resonance

To determine the potential for Q2_TFL_ as a noninvasive ^19^F MR dynamic probe, initial ^19^F NMR was performed on a 500 MHz NMR spectrometer. Q_TFL_ and Q2_TFL_ exhibited triplet NMR peaks (Figure S10a, b) consistent with the triple fluorinated residue motif. Due to the presence of peak overlap in the spectrum of Figure S10a, b, the accurate identification and distinction of individual peaks becomes challenging. Specifically, peak 3, characterized by the largest chemical shift, overlaps with peak 2, making it difficult to reliably detect and distinguish them. We attribute this overlap and reduced clarity to protein conformational heterogeneity, which can result in line broadening. This overlap hinders the clear resolution of the individual contributions of these peaks, potentially complicating their proper identification and quantification. Despite the challenges posed by the peak overlap, we were able to characterize the overall *T*_1_ and *T*_2_ relaxation times of Q2_TFL_ in its ^19^F NMR spectrum. Q2_TFL_ demonstrated a ^19^F *T*_1_ relaxation time of 329 ms and a *T*_2_ relaxation time of 120 μs in its ^19^F NMR spectrum at 25 °C and 5.6 mg/mL. In comparison, previous findings from our group reported that F-TRAP at a concentration of 1 mg/mL and 22 °C, exhibited ^19^F *T*_1_ of 393 ms and a *T*_2_ of 1.2 ms^31^ suggesting the increased rigidity of Q2_TFL_.

**Figure 4:**
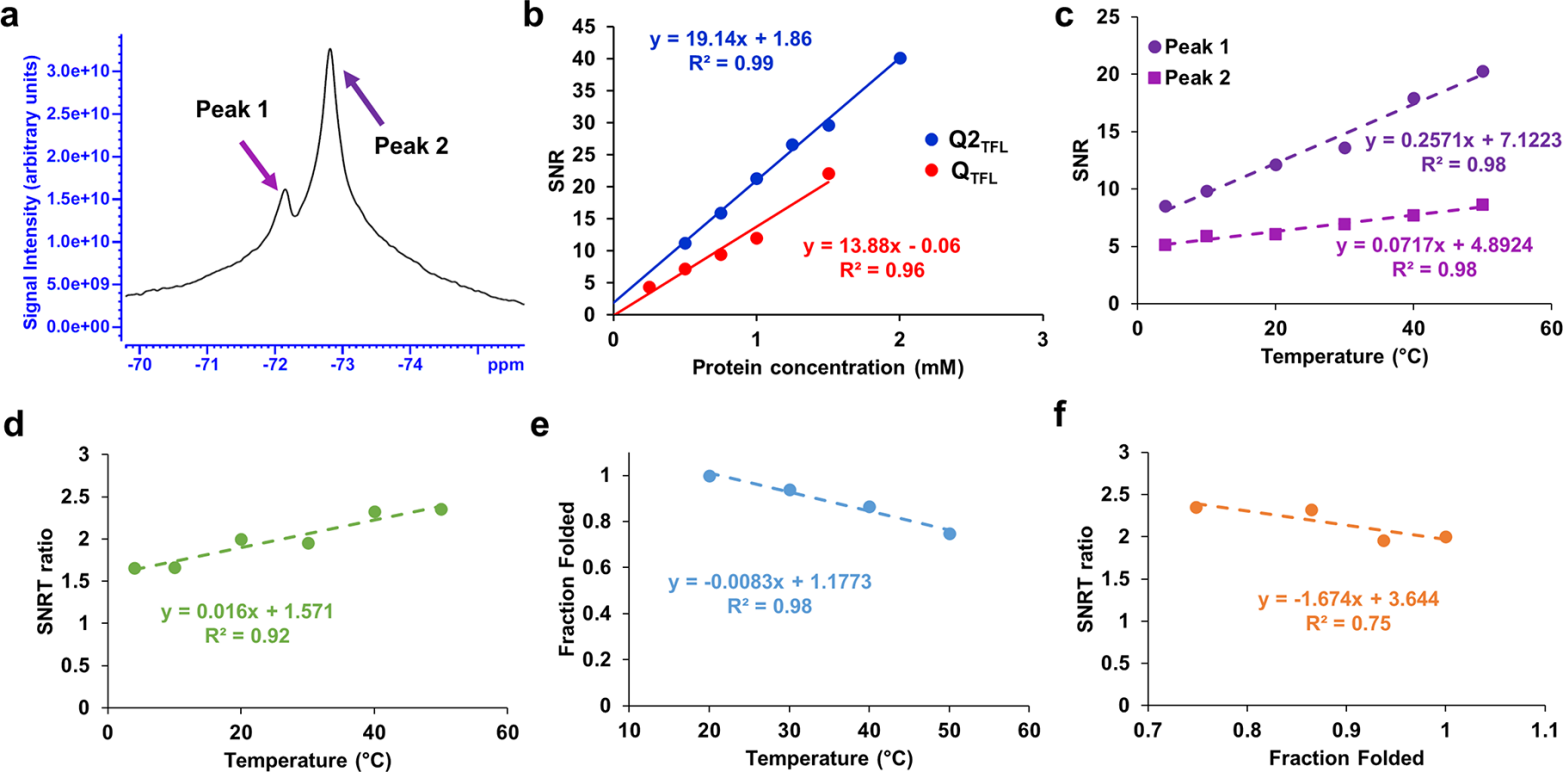
(a) NMR spectrum at 500 MHz (11.7-T) of Q2_TFL_ at 1.5 mM shows two peaks (magenta and purple arrows), (b) SNR of Q2_TFL_ and Q_TFL_ as a function of protein concentration. (c) Temperature dependence of SNR from independent peaks. (d) Linear correlation of temperature with SNRT ratio showing the ability to predict temperature from ^19^F MRS. (e) Linear correlation of temperature with average (*n* = 3) fraction folded of Q2_TFL_ as assessed by CD. (f) Linear correlation of average fraction folded (*n* = 3) as assessed by CD with SNRT ratio showing ability to predict relative structure from ^19^F MRS.

Q2_TFL_ and Q_TFL_ were measured at molar concentrations 0.25–2.0 mM (Table S6) and the signal-to-noise ratio (SNR) was measured for each spectrum using 50–100 ppm to represent all signals that appeared in the spectra and 0–50 ppm, where no signal appeared, to represent the noise. TFA exhibited a chemical shift of −76.1 ppm (Figure S10a), consistent with reported values.^63^ Q2_TFL_ displayed a chemical shift of −72.8 ppm (Figure 4a, Figure S10b), whereas parent Q_TFL_ exhibited a chemical shift of −72.6 (Figure S10c). Additionally, Q2_TFL_ demonstrated a SNR dependence on the ^19^F molar concentration of 19.14 mM^–1^, while Q_TFL_ showed a relationship of 13.88 mM^–1^ to SNR (Figure 4b, Figure S11). Notably, the SNR efficiency with respect to the ^19^F molar concentration of Q2_TFL_ was 1.38 times greater than that of Q_TFL_, which is consistent with the expected increase based on the theoretical 9/7 TFL ratio of Q2_TFL_/Q_TFL_.

In comparison, our previous fluorinated construct, F-TRAP, exhibited an SNR efficiency with respect to ^19^F molar concentration of ∼13.6 mM^–1^ at a magnetic field strength of 400 MHz.^31^ To account for the difference in magnetic field strength *B*_0_, we conducted an estimation of SNR performance at 500 MHz, based on its well-established dependence on the static magnetic field strength, .^45,64^ Using this relationship, our projection indicates that F-TRAP could achieve an SNR performance of ∼19.0 mM^–1^ at 500 MHz. This estimation suggests that Q2_TFL_, as an improved protein-engineered drug delivery agent, generates a stronger ^19^F MR signal at equal molar concentrations compared to our previously detectable ^19^F MR biomaterial, F-TRAP.^31^ Furthermore, Q2_TFL_ possesses 9 TFL per monomer with a monomeric molecular weight of 6.97 kDa, whereas F-TRAP has 11 TFL per monomer with a monomeric molecular weight of 16.74 kDa. This translates to an SNR slope of 2.74 mg/mL^–1^ for Q2_TFL_, which is 2.4 times higher than the 1.14 mg/mL^–1^ SNR slope for F-TRAP. These results suggest that Q2_TFL_ is significantly more powerful by mass.

Finally, Q2_TFL_ was assessed for temperature dependence by altering the environmental temperature in NMR. Q2_TFL_ exhibited an increase in SNR with an increase in temperature, dominated by peak 2 at all temperatures. SNR of each peak was assessed individually by acquiring 1 ppm breadths around peaks 1 and 2 resulting in independent temperature coefficients (Figure 4c). At constant concentration, the ratio of these slopes was used to determine an independent SNR-temperature coefficient for Q2_TFL_ dubbed the SNRT ratio. As expected, linear temperature dependence was retained with the SNRT ratio (Figure 4d), illustrating the intuitive capability to predict temperature using the ratio of peaks 1 and peak 2. This suggested that it could serve as a valuable tool for temperature monitoring. Furthermore, Q2_TFL_ possessed a linear correlation at *in vivo* relevant temperatures with an R^2^ = 0.98 (Figure 4e). Thus, the SNRT ratio was correlated with the fraction folded assessed by CD at *in vivo* relevant temperatures with an *R*^2^ = 0.75 (Figure 4f), indicating a strong linear relationship and demonstrating the ability to predict relative structure from overall SNRT ratio alone. These preliminary results show promising potential for the applications of SNRT. Further investigations can explore its use as a valuable tool for *in vivo* monitoring of Q_TFL_ structure and temperature, particularly in areas such as hyperthermia and drug release control. However, conducting *in vivo* experiments and exploring these applications require additional research and careful considerations. This study serves as an initial step toward these possibilities.

### Phantom Magnetic Resonance Imaging

**Figure 5:**
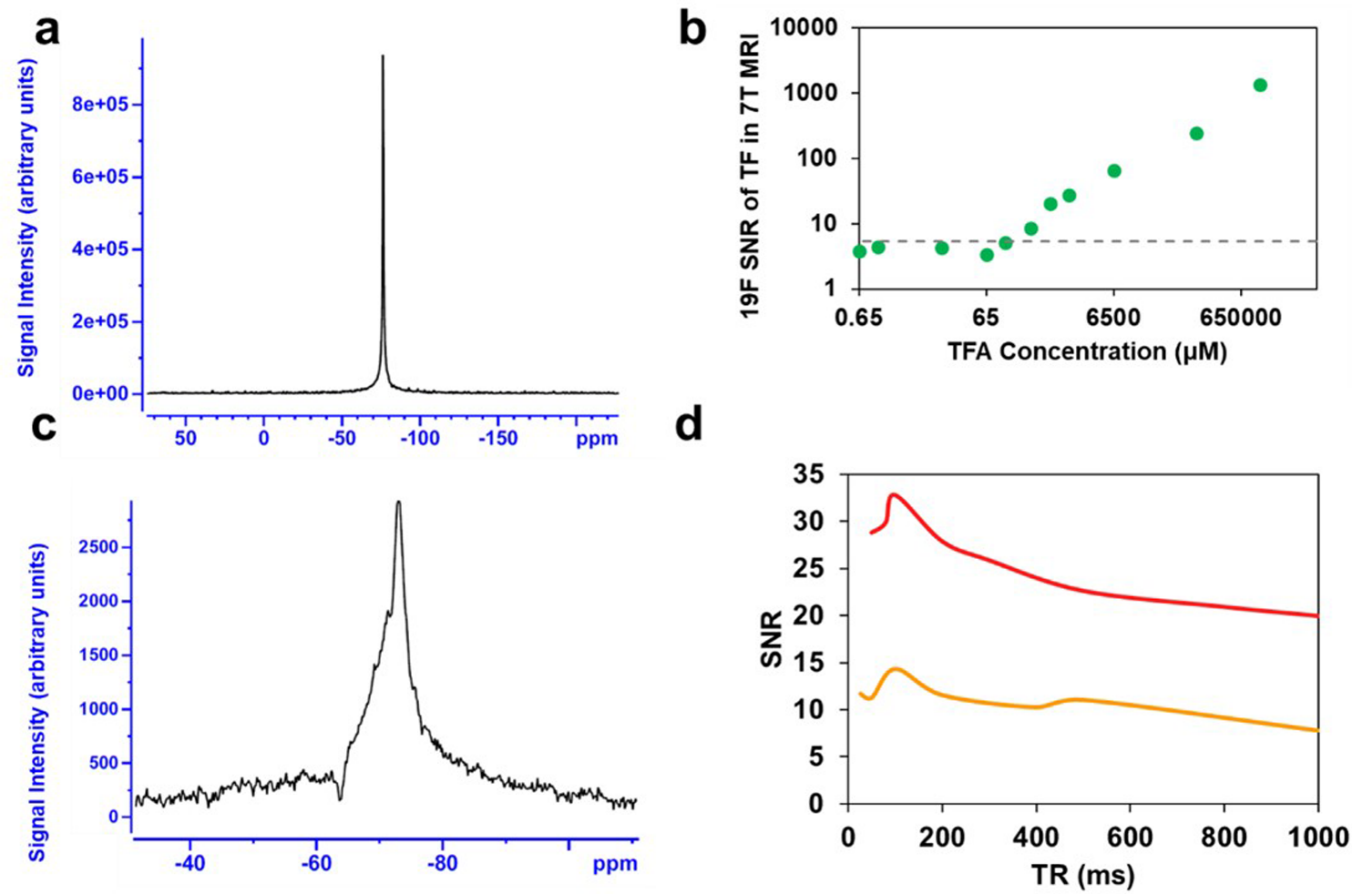
(a). Representative in vitro TFA spectra (100%, 13 mM) were acquired using our experimental setup and custom RF coil on a 7-T animal MRI scanner (300 MHz) employing a single pulse sequence. (b) Corresponding ^19^F SNRs at 7-T MRI are presented for serial dilutions of 100% TFA (green), progressively diluted until reaching the limit of detection (LOD) threshold indicated by dashed lines. (c) Representative ^19^F MR scan (scan time = 4 min, TR = 80 ms). (d) SNR of Q2_TFL_ obtained from ^19^F MRS using a 7-T MRI scanner, with scans acquired under both 4 min (red) and 1 min (orange) scan times, while varying the TR.

Q2_TFL_’s potential as a traceable drug delivery agent was assessed through *in vivo* experiments conducted with our customized rf coil specifically designed for the 7-T Bruker 7030 Biospec μ-MRI system. This single-port dual-resonance (^1^H/^19^F) rf coil was tailored to provide optimal coverage of body extremities, such as the knees, during the experiments. The MRI sequence parameters were first optimized by phantom imaging of 100 μL of 100% TFA (13 mM) and 1 mM Q2_TFL_ samples. The limit of detection (LOD) (Figure 5a) was assessed for ^19^F using TFA (Figure 5b) with a spectral resolution of 41.6 Hz/pt. The threshold for the LOD calculation was achieved at a SNR of 5.3, which is equal to three standard deviations above the baseline noise level. This threshold was reached at 130 μM TFA. Based on the relative SNR of Q2_TFL_, this suggests that the LOD would be reached by ∼100 μM Q2_TFL_ using the 1.46 Q2_TFL_:TFA (mM:mM) SNR ratio as determined by NMR.

Prior to *in vivo* studies, we aimed to optimize the SNR of Q2_TFL_ in ^19^F MRS by finding a balance between the length of the scan time and the repetition time (TR). Here, we varied TR using a shorter scan time (1 min) and a longer scan time (4 min). To do so, the number of averages was adjusted to maintain a consistent scan time across different TR values (Figure 5c, Table S7). Here, we empirically optimize the performance of our pulse sequence parameters while adhering to a fixed imaging time frame, where 4 min scans were used for static studies and 1 min for dynamic studies (which aided us in identifying potential overlap with fluorinated anesthetics, such as isoflurane). Notably, Q2_TFL_ exhibited the highest signal performance at TR between 80 and 100 ms. Longer scan time of 4 min (Figure 5d) showed a significant improvement in SNR, while shorter 1 min scan times yielded an expected ∼2× reduction in SNR. Nevertheless, the sensitivity of the 1 min scan remained above the LOD, allowing for the acquisition of Q2_TFL_ spectra with improved temporal resolution for traceability purposes. Overall, these studies allowed us to determine a suitable balance between scanning parameters: TR and number of averages.

### *In Vivo* Magnetic Resonance Imaging

**Figure 6:**
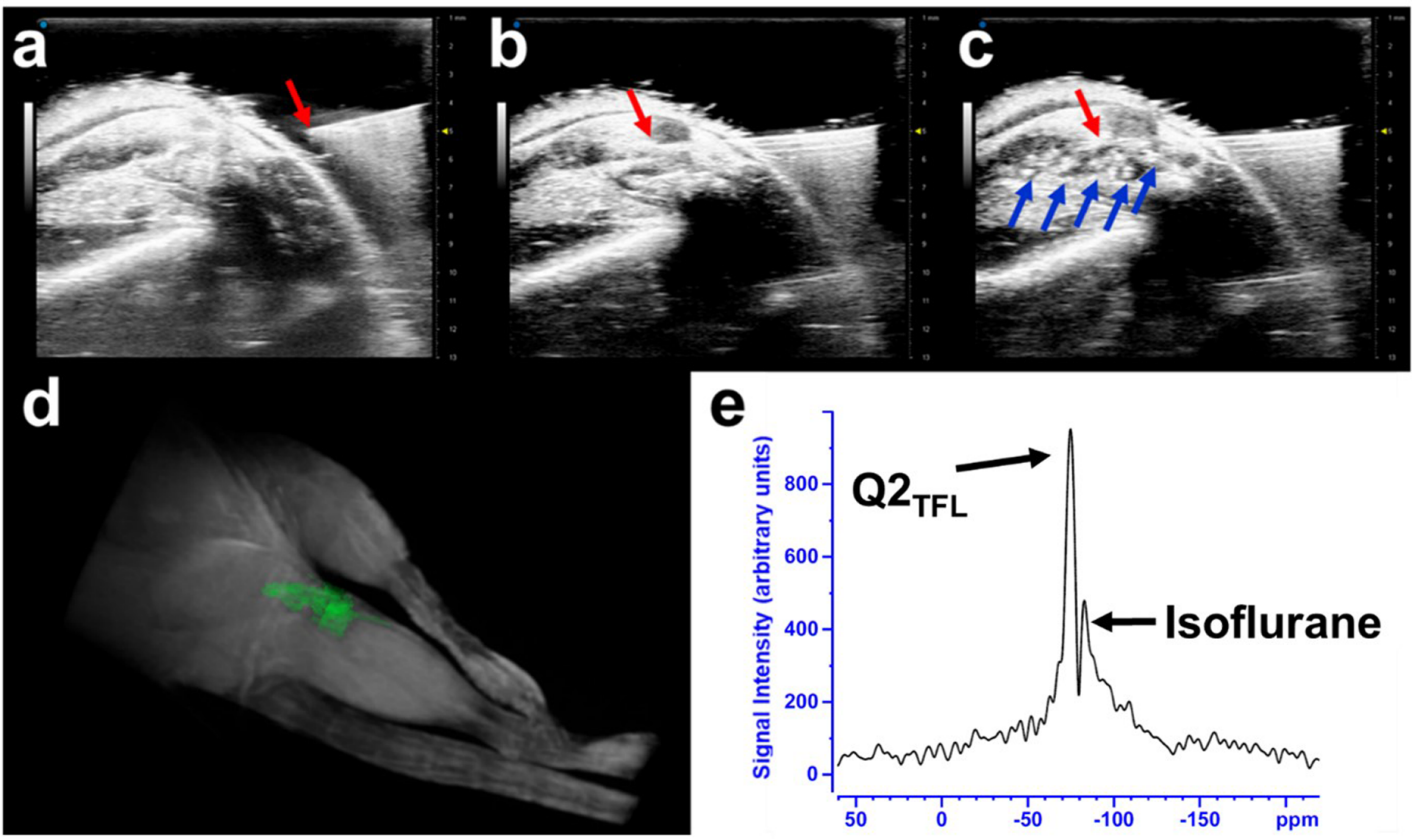
Ultrasound guided injection imaging: (a) Sagittal view of the hindlimb right before the needle insertion which is adequately tilted at 45° to expose to joint and ease the infusion. (b) The needle insertion within the hindlimb knee joint. (c) Successful injection of Q2_TFL_ into the hindlimb knee joint appearing as an echogenic signal using high frequency ultrasound. Red arrow indicates the syringe tip, and blue arrows indicate the presence of Q2_TFL_. (d) 3D rendering of ^1^H MRI imaging of the mouse hindlegs where Q2_TFL_ fibers (highlighted in green) appeared as a hypointense signal in the 3D MRI data sets in the injected hindleg. (e) ^19^F MR spectroscopy performed *in vivo* after injection of Q2_TFL_ using 10 min scan (TR = 100 ms, *N*_AV_ = 6000).

4-to-6-week-old C57Bl6 mice were used to demonstrate the *in vivo*^1^H MRI and ^19^F MRS traceability of Q2_TFL_. Mice were intra-articularly injected with a 50 μL volume of 1 mM Q2_TFL_ protein, guided by ultrasound (Figure 6a–c). Consistent with our recent work using a coiled-coil fusion protein to target disease prevention in osteoarthritis,^65^ we use the knee joint as model for localized injection, where here we focus on imaging modalities of Q2_TFL_. Throughout the imaging experiments, the Q2_TFL_ fibers appeared immobilized using both high frequency ultrasound and MRI. Notably, Q2_TFL_ revealed high frequency echogenic properties as shown using a phantom setup (Figure S12) and *in vivo* experiments (Figure 6a–c).

Three-dimensional gradient echo (3D-GE) imaging of mouse hindlimbs was conducted under 200-μm isotropic resolution (Figure 6d). The images revealed the presence of Q2_TFL_ in the injected joint, observed as a hypo-signal on MRI due to its short *T*_2_ transverse relaxation time (Figure S13). *T*_2_-shortening of Q2_TFL_ could be attributed to the semisolid fibers, which provide rigidity and result in dipolar interactions within the protein.^66^ Additionally, the high protein concentration creates a hydrophobic environment, restricting water mobility and further contributing to the observed hypo-signal.^67,68^

*In vivo*^19^F MR spectroscopy showed a chemical shift of −72.8 ppm (Figure 6e) corresponding to Q2_TFL_ with a SNR of 20.6. Interestingly, the spectra also revealed a neighboring peak with a chemical shift of −78.0 ppm, which we attribute to the use of isoflurane as an inhaled anesthetic during *in vivo* mouse imaging. This was verified by turning off isoflurane while performing a series of 1 min scans over time. As respiration increased due to clearance of the anesthetic, the SNR of the peak at −78.0 ppm gradually decreased, while the Q2_TFL_ SNR remained stable (Figure S14). This observation provides further evidence supporting the identification of the peak at −86.7 ppm as a result of the isoflurane presence in the spectra.

Finally, a comparative analysis was conducted to assess the relative SNR of Q2_TFL_*in vivo* compared to the previous fluorinated construct, F-TRAP.^31^*In vivo*^19^F MRS was performed using the same sequence timing as used for F-TRAP, while ensuring optimized conditions for Q2_TFL_ pulse sequence parameters (TE, TR, *N*_AV_) and MRI coil. The scan consisted of 4000 averages with a TR of 100 ms, resulting in a total scan time of 6 min and 40 s. The obtained SNR for Q2_TFL_ was 11.45 using 7.0 mg/mL (corresponding to 1 mM Q2_TFL_ and 9 mM ^19^F) (Figure S15). The results demonstrated a substantial improvement in the SNR of Q2_TFL_, in terms of both weight (2.0×) and mM yield of ^19^F (2.5x), which can be attributed to a higher ^19^F-protein ratio and monomer density in the fiber morphology, leading to stronger ^19^F packing. The relatively short *T*_2_ of Q2_TFL_ indicates that signal enhancement may be further improved via sequence optimization. Nevertheless, the notable SNR already observed in the ^19^F MRS holds promise for the application of Q2_TFL_ as an imaging agent.

We have demonstrated Q2_TFL_ to possess bimodal mapping through echogenicity for high-frequency ultrasound visualization (Figure 6, Figure S12), and T2-darkening MRI contrast relative the surround tissue (Figure 6 and Figure S15) while also being traceable by ^19^F MRS. To the best of our knowledge, this is the first protein fiber to have the capability of a multimodal imaging agent. Furthermore, Q2_TFL_ exhibited utility as a probe for both environmental temperature and protein structure analysis. In addition to its encapsulation ability, which increased thermostability and thickness, these attributes represent a foundation for the future development of biomaterials that possess novel theranostic behavior.

## Conclusions

Q2_TFL_ forms fibers on the nano- to mesoscale and generates a larger increase in thermostability and SNR compared to our previously fluorinated fiber construct, Q_TFL_, at the same concentration, demonstrating its ability for ^19^F magnetic resonance detection. Furthermore, Q2_TFL_’s therapeutic potential in the form of drug delivery has been demonstrated by its ability to encapsulate CCM. We further explore its ability to thicken and thermostabilize upon CCM binding, as well as its stimuli-responsiveness to ionic strength. Processing of TFL triplet behavior in Q2_TFL_ potentially allows for additional function as a temperature probe and monitoring of the relative protein structure of the agent. Finally, we demonstrate the ability of Q2_TFL_ to provide multimodal contrast in both ^1^H MRI and high frequency ultrasound with sensitive traceability by ^19^F MRS *in vivo*. The results here provide important criteria toward fluorination of coiled-coils for supramolecular assembly and design toward ^19^F MRS theranostic agents. These results provide a foundation for future *in vivo* investigations in this area and to explore the potential applications of Q2_TFL_*in vivo*.

## Supporting Information Available

Figures of experimental MR setup designed in-house, Q_TFL_ expression and purification, representative Q_TFL_ ATR-FTIR spectra, MALDI-TOF spectra for Q2_TFL_ and Q_TFL_ calculations, NMR spectra of Q_TFL_ and TFA, representative TEM images of Q2_TFL_, representative fluorescent confocal micrographs of CCM-bound Q2_TFL_, model of CCM-bound fiber diameters compared to native fiber diameters and relative placement of Q2_TFL_, *in vivo*^19^F MR spectra of Q2_TFL_ at 6 min 40 s scan time, Q2_TFL_ phantom echogenicity, Q2_TFL_ spectroscopy over time after turning off fluorinated anesthetic, and Tables for secondary structure compositions by CD and ATR-FTIR deconvolution, TFL incorporation calculation results for independent trials of Q_TFL_ and Q2_TFL_, SNR values for Q2_TFL_ and Q_TFL_ by NMR, and SNR values for Q2_TFL_ at varying TR (PDF)
