# Supplementary material for: Protein-Engineered Fibers For Drug Encapsulation Traceable via 19F Magnetic Resonance
Source: ACS Appl Nano Mater. 2023 Nov 6;6(22):21245–57. doi: 10.1021/acsanm.3c04357 (PMC10682962; doi:10.1021/acsanm.3c04357)
Supplement: Supplementary file 1 — an3c04357_si_001.pdf [file an3c04357_si_001.pdf]

Supporting Information

Protein-engineered Fibers For Drug Encapsulation Traceable *via* ^{19}F Magnetic Resonance

Dustin Britton[†], *Jakub Legocki*[†], *Orlando Aristizabal*[§,¶], *Orin Mishkit*[§,¶], *Chengliang Liu*[†], *Sihan Jia*[†], *Paul Douglas Renfrew*[∥], *Richard Bonneau*[∥,⊥,#], *Youssef Z. Wadghiri*[§,¶], *and Jin Kim Montclare*[†,¶,∇,○*]

† Department of Chemical and Biomolecular Engineering, New York University Tandon School of Engineering, Brooklyn, New York, 11201, USA

§.Center for Advanced Imaging Innovation and Research (CAI2R), New York University School of Medicine, New York, New York, 10016, USA

¶.Bernard and Irene Schwartz Center for Biomedical Imaging, Department of Radiology, New York University School of Medicine, New York, New York, 10016, USA

∥. Center for Computational Biology, Flatiron Institute, Simons Foundation, New York, New York, 10010, USA

⊥. Center for Genomics and Systems Biology, New York University, New York, New York, 10003, USA

#. Courant Institute of Mathematical Sciences, Computer Science Department, New York University, New York, New York, 10009, USA

∇ Department of Chemistry, New York University, New York, New York, 10012, USA

° Department of Biomaterials, New York University College of Dentistry, New York, New York, 10010, USA

* Corresponding author

Email: montclare@nyu.edu and youssef.zaimwadghiri@nyulangone.org

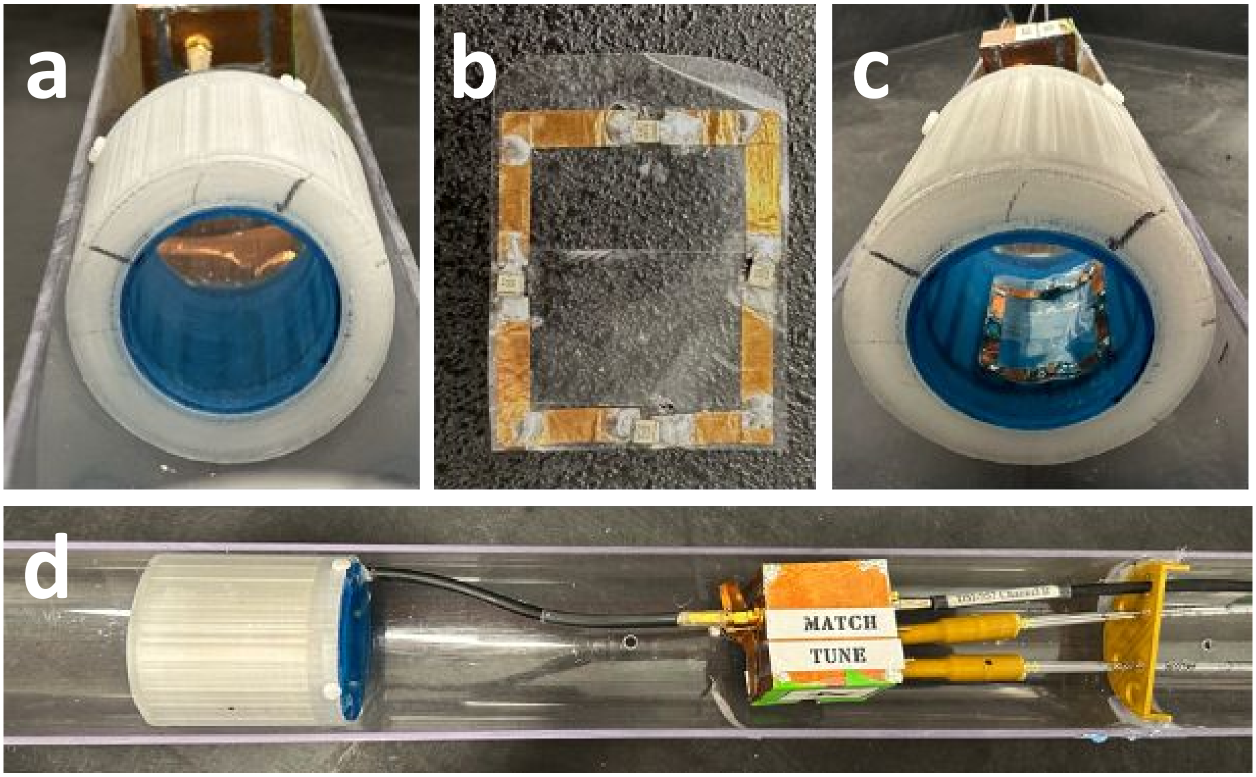

Figure S1. a. Transmit-receive volume linear birdcage radiofrequency (rf) coil (16 rungs, OD=72mm, ID=42mm, L= 64mm) tuned to resonate at 300.16 MHz, corresponding to the ^{1}H proton Larmor frequency at 7-Tesla. It ensures rf coverage for the entire mouse body. **b.** Rectangular flexible rf resonator (L=10mm, W=30mm) tuned the ^{19}F nuclei (282 MHz) at 7-T using four distributed fixed capacitors to electrically balance the resonator. **c.** The flexible rf surface coil is positioned within the inner part of the cylindrical birdcage to optimize inductive coupling enabling dual ^{1}H/^{19}F resonance via a single port interfaced to a tune/match box. **d.** Illustration of the experimental setup enabling *in vivo* mouse MRI and MRS scanning of the mouse body with coverage optimized for the extremities such as the knees.

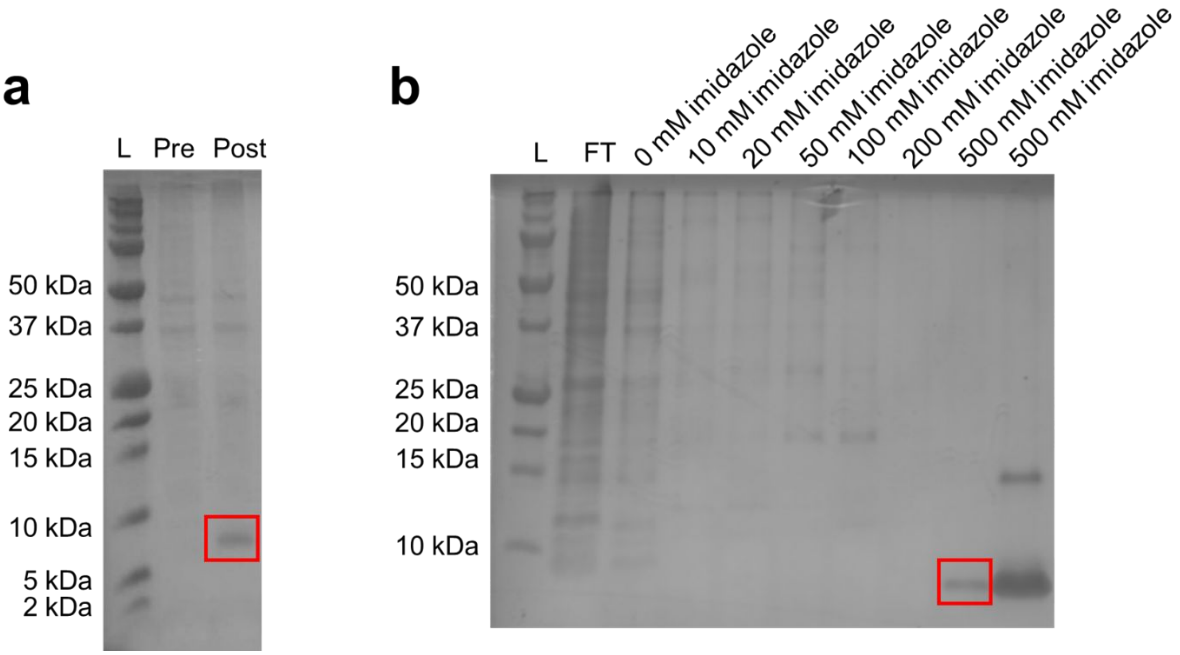

Figure S2. a. Q_{TFL} protein (6.80 kDa) after expression. L; Ladder, Pre: Pre-induction with IPTG, Post: Post-induction with IPTG. **b.** Q_{TFL} protein (6.80 kDa) after purification. L: Ladder, FT: Flow-through, following are increasing concentrations of imidazole.

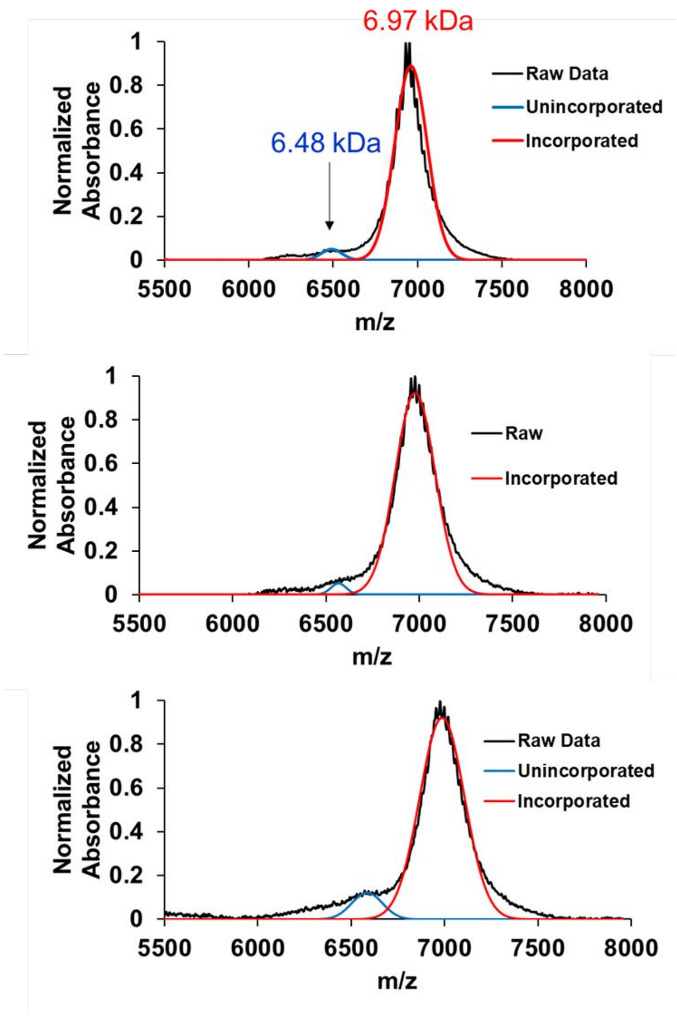

Figure S3. All MALDI-TOF spectra used in calculation of average TFL incorporation for $Q2_{TFL}$.

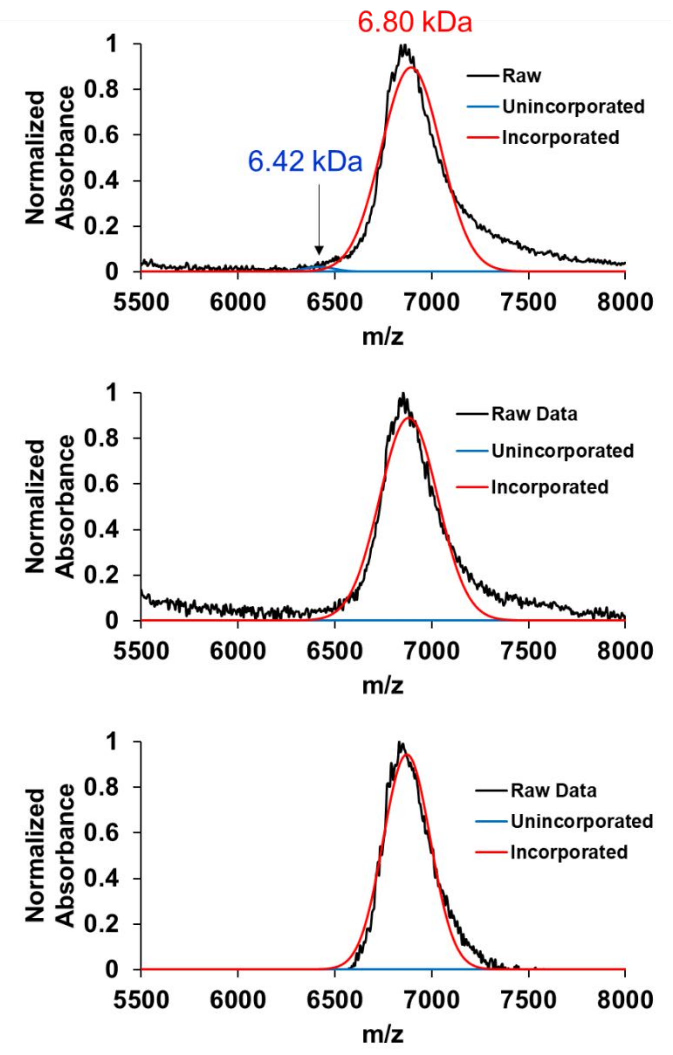

Figure S4. All MALDI-TOF spectra used in calculation of average TFL incorporation for Q_{TFL}.

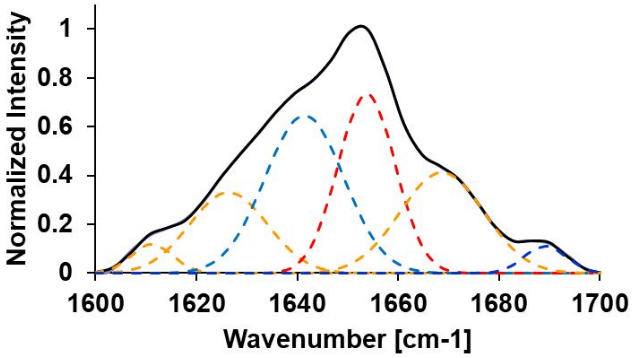

Figure S5. Representative ATR-FTIR spectrum of Q_{TFL}. Overall spectrum by deconvolution in black and individual peak deconvolutions in dotted red lines (α-helix), blue lines (β-sheet), and orange lines (random coil/turns).

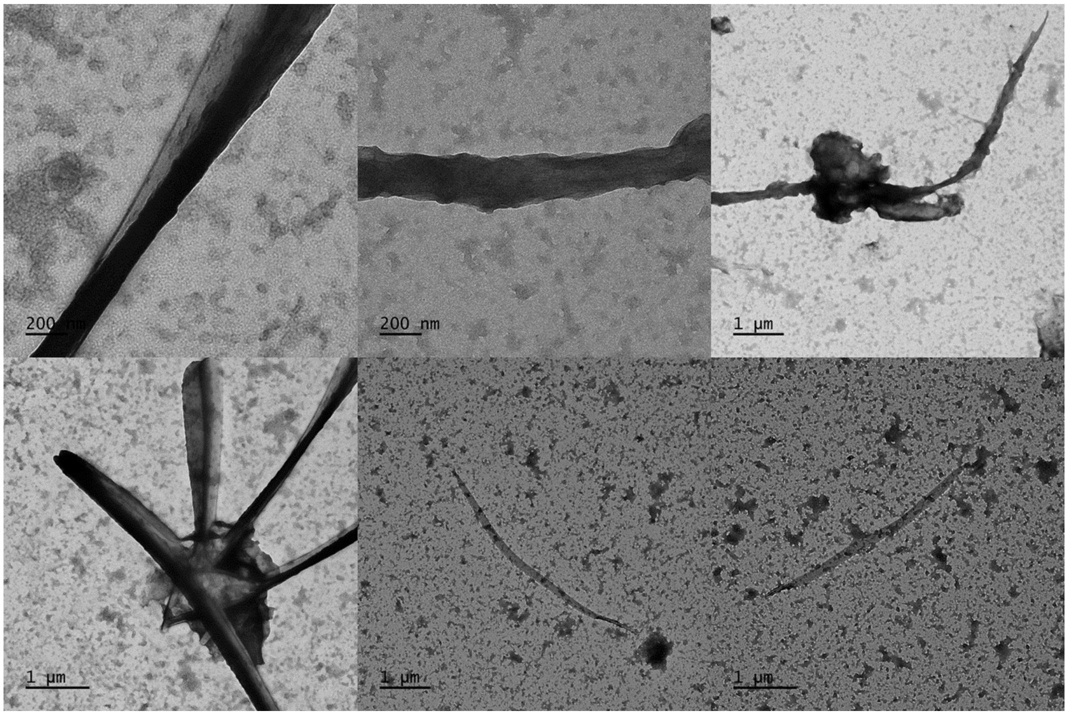

Figure S6. Various resolution TEM images of $Q2_{TFL}$.

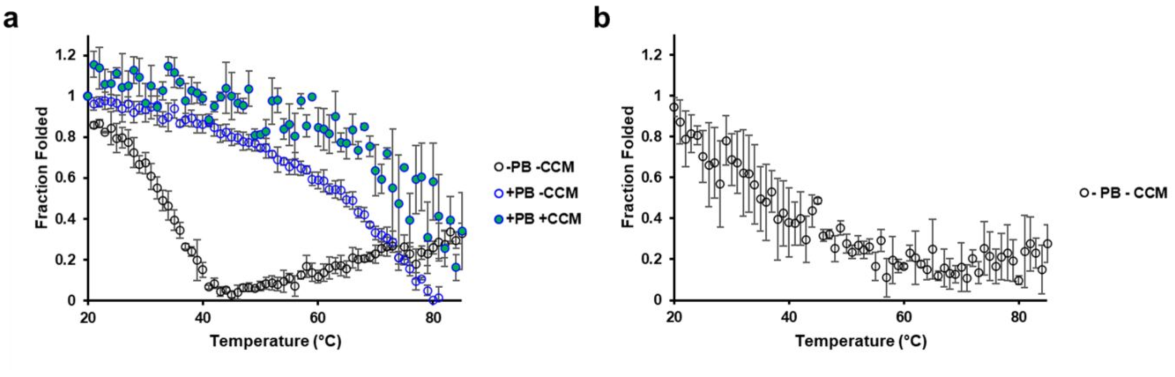

Figure S7. a. Average fraction folded of **a.** Q2$_{TFL}$ and **b.** Q$_{TFL}$ in the presence of with (+) and without (-) phosphate buffer (PB) and curcumin (CCM). Error bars are the standard error of three independent trials.

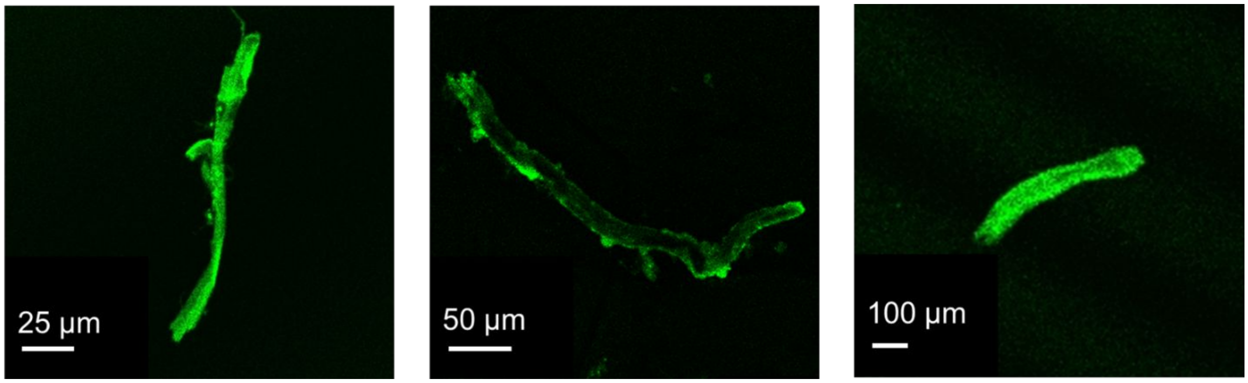

Figure S8. Fluorescent confocal micrographs of Q2$_{TFL}$ at various resolutions

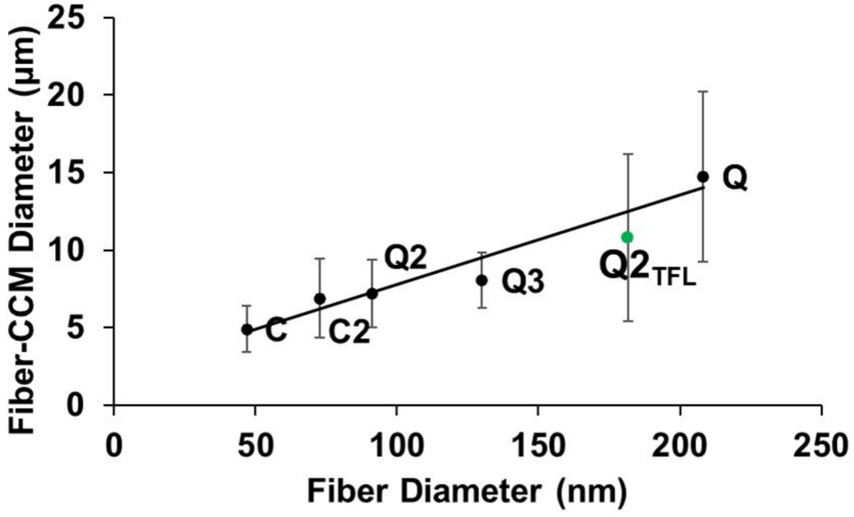

Figure S9. CCM-bound fiber diameters measured by confocal microscopy compared to fiber diameters measured by TEM. Black dots represent previous fiber constructs and black line represents previous linear relationship. Green dot represents Q2$_{TFL}$. Error bars represent standard deviation of 20 fiber diameters.

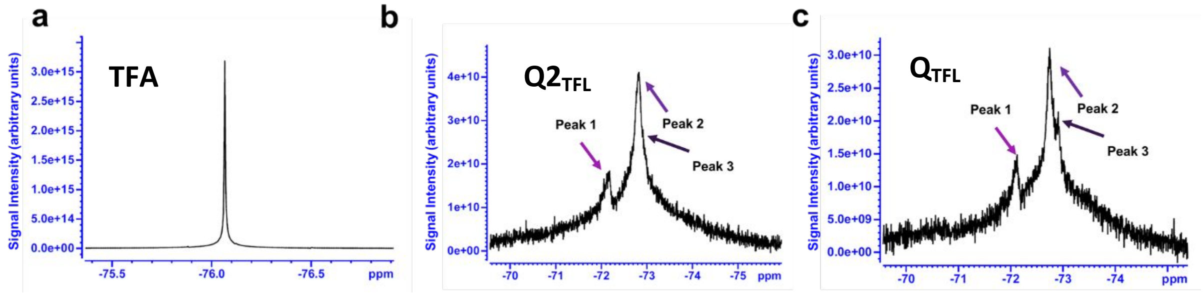

Figure S10. NMR spectra acquired 500 MHz for **a.** TFA (11.8 mM) in 10% D_2O **b.** 1.5 mM $Q2_{TFL}$ and **c.** 1.5 mM Q_{TFL} without line broadening and with arrows indicating ^{19}F peak signals 1-3 locations. Spectra acquired using 256 scans.

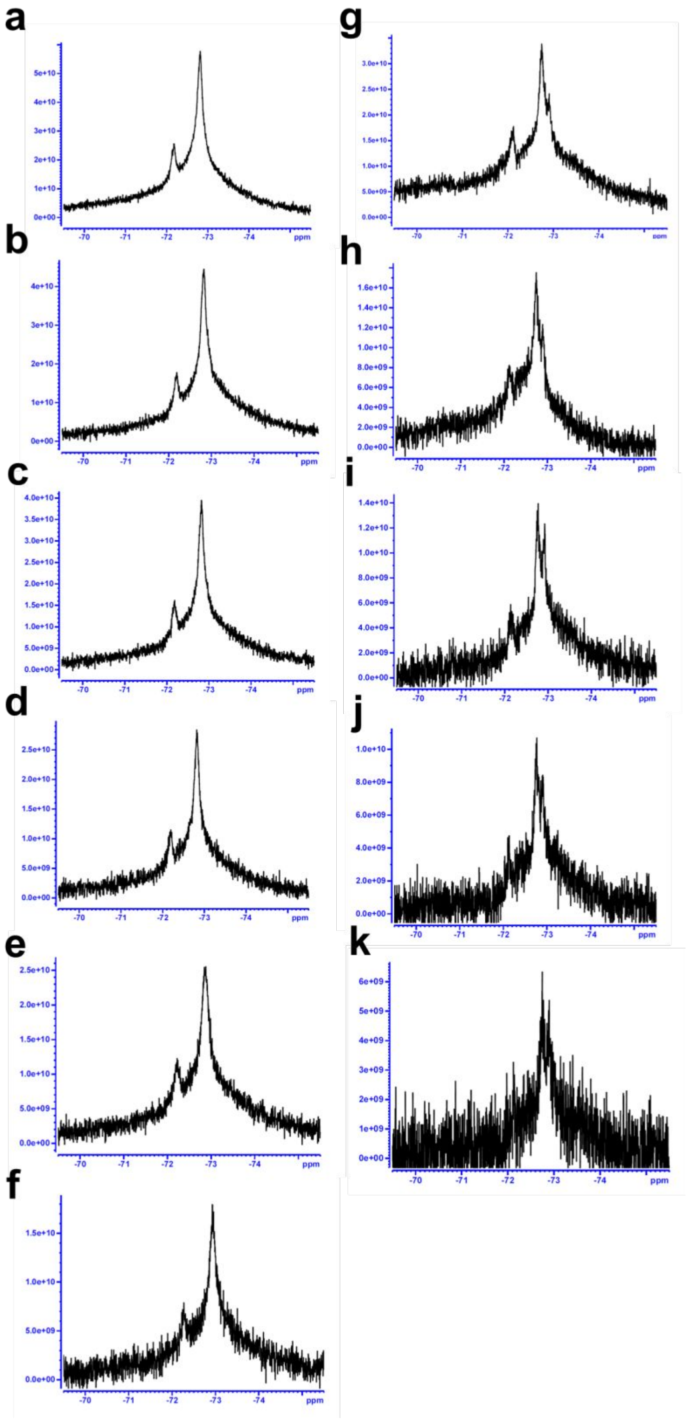

Figure S11. NMR spectra acquired 500 MHz for Q_{2TFL} **a.** 2.0 mM **b.** 1.5 mM **c.** 1.25 mM **d.** 1.0 mM, **e.** 0.75 mM **f.** 0.5 mM and Q_{TFL} **g.** 1.5 mM **h.** 1.0 mM **i.** 0.75 mM **j.** 0.5 mM and **k.** 0.25 mM.

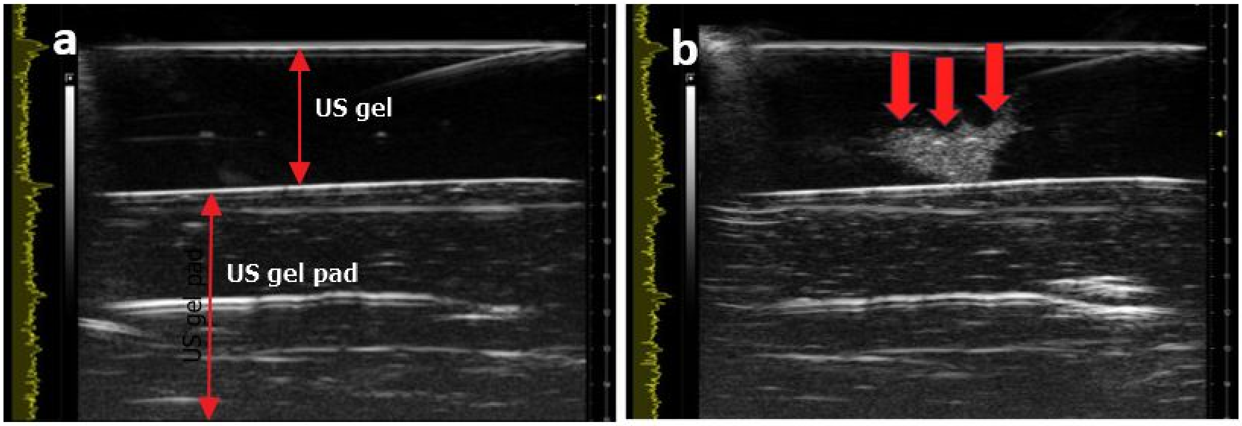

Figure S12. The acoustic backscatter property of $Q2_{TFL}$ was investigated using an ultrasound phantom with the Vevo 3100 high-frequency ultrasound scanner (VisualSonics - Fujifilm). The ultrasound images were acquired using the MX550D 40 MHz transducer. Ultrasound gel (Aquasonic Clear, Parker laboratories,Fairfield, NJ) was applied on top of an ultrasound gel pad (Aquaflex, Parker laboratories,Fairfield, NJ) and the face of the transducer was lowered until touching the US gel. **a.** The B-mode image of the ultrasound gel shows an anaechoic region between the face of the transducer and the gel-gel pad interface. **b.** After pipetting the $Q2_{TFL}$ into the US gel, B-mode image clearly demonstrates the highly echogenic signal from $Q2_{TFL}$ (red arrows) resulting in ultrasound image contrast.

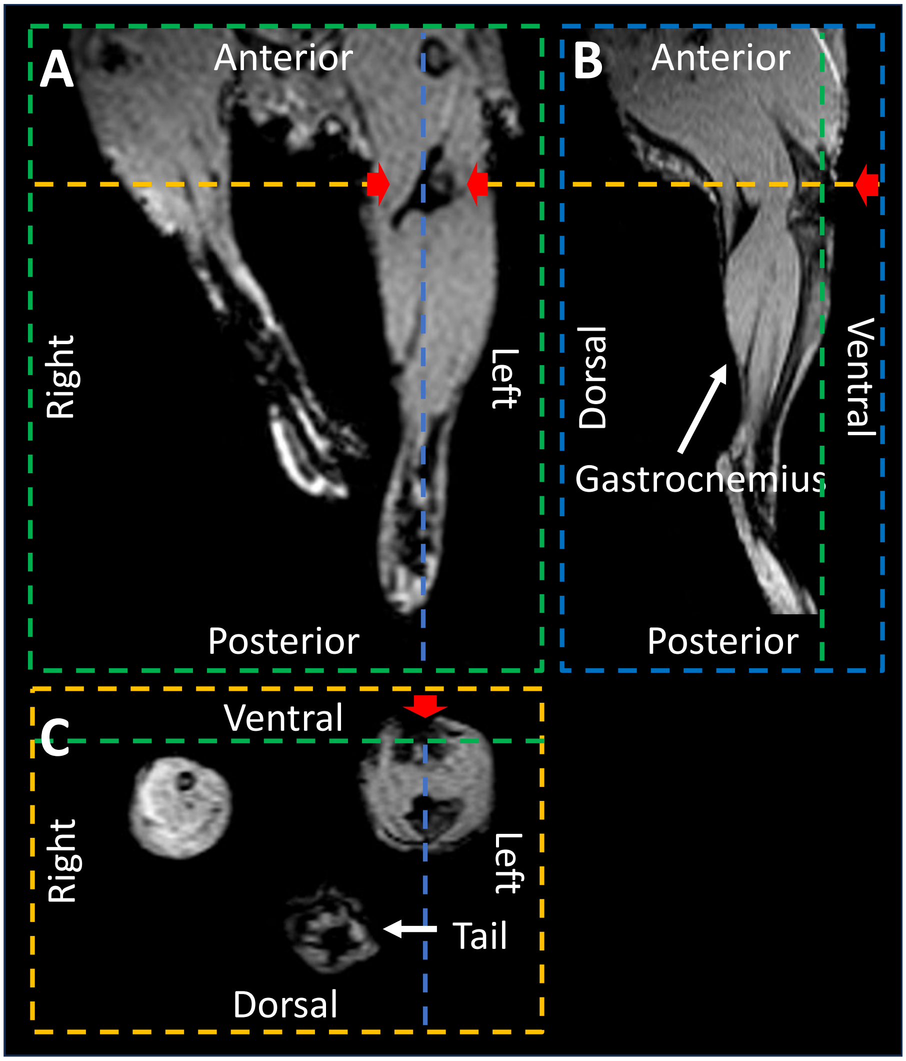

Figure S13. The 3D ^{1}H MRI dataset covering the lower region of the mouse's body provides a clear visualization of anatomical structures from multiples perspectives, presented in three anatomical orientations, thanks to the 200 µm isotropic resolution. Within this dataset, the presence of the $Q2_{TFL}$ fibers, which were injected into the joint of the left hind leg, is readily apparent, as indicated by the red arrows. This distinctive presence is characterized by a pronounced signal void, significantly facilitating the precise delineation of its spatial extent. Furthermore, the 3 cross-sections effectively highlight the hypointense contrast exhibited by the $Q2_{TFL}$ fibers, further emphasizing their distinct visibility within the ^{1}H MRI dataset. To provide a more comprehensive depiction of this observation, we have included a 3D rendering showcasing the distribution of the $Q2_{TFL}$ fibers in **Figure 6D**. These findings collectively underscore the effectiveness of $Q2_{TFL}$ as a hypointense contrast agent in ^{1}H MRI, yielding a discernible and valuable hypo-signal, which in turn allows for the precise localization and mapping of its distribution within the anatomical context.

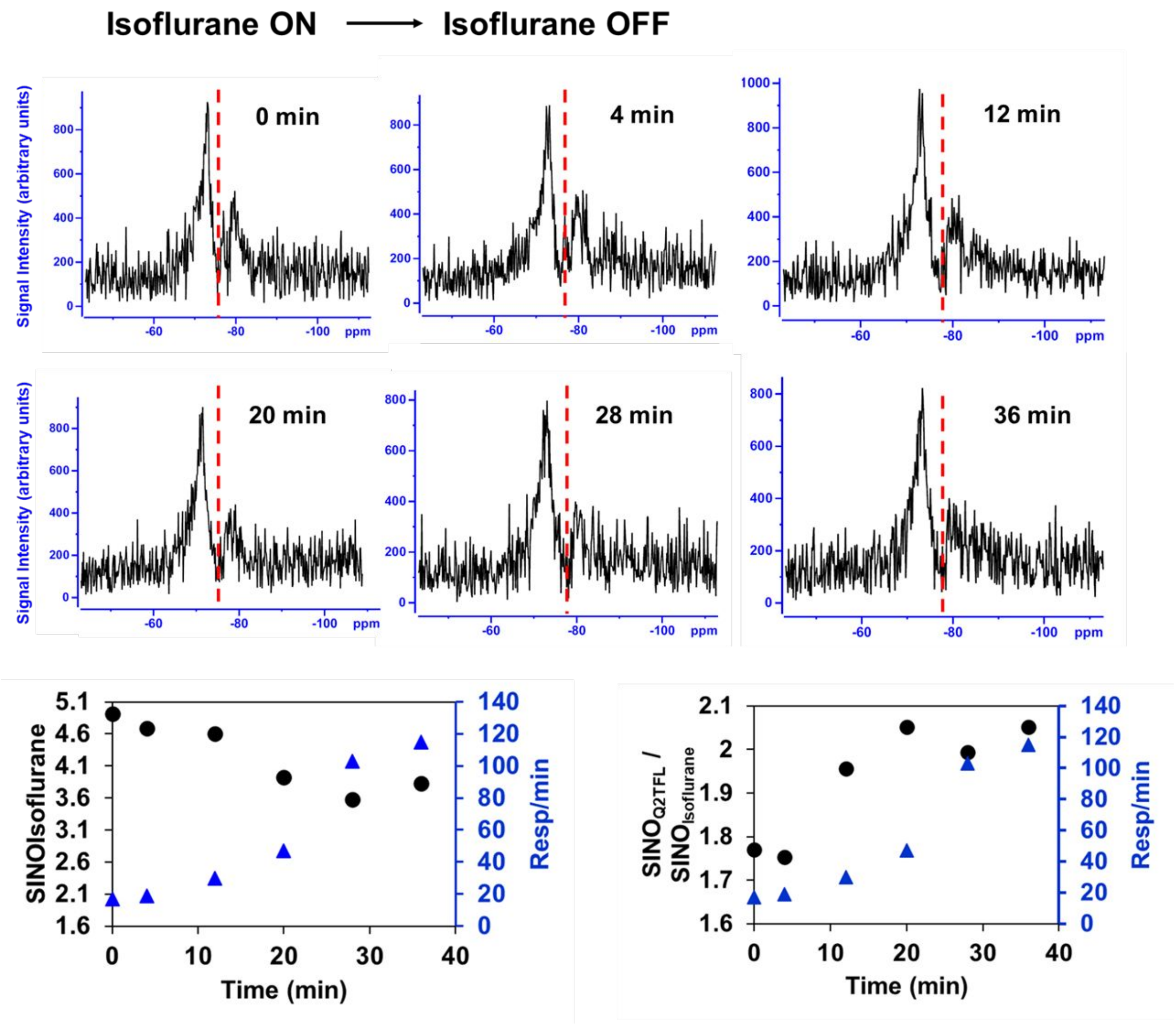

Figure S14. *In vivo* ^{19}F MR spectra with $Q2_{TFL}$ injected within the joint after turning off the administration of isoflurane as an anesthetic. The resulting SNR of the isoflurane peak and the ratio of SNR between $Q2_{TFL}$ peak and isoflurane peak overtime in comparison to respirations/min of the mouse. The red dashed line shows the point of separation used to distinguish $Q2_{TFL}$ and isoflurane peak in SNR calculations.

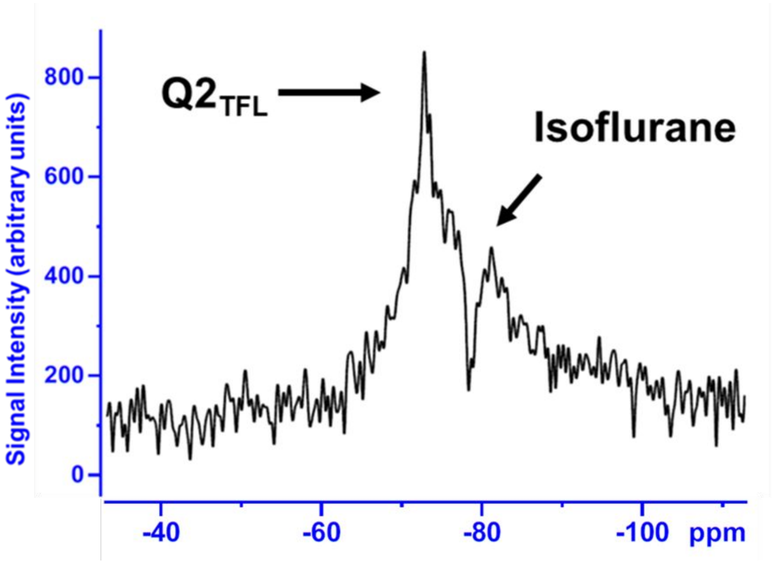

Figure S15. *In vivo* ^{19}F MR spectra using 6 m 40 s scan time at 100 ms TR of $Q2_{TFL}$.

Table S1. Sequences for fluorinated and non-fluorinated Q and Q2 constructs and calculated molecular weights.

Protein	Sequence	MW (kDa)
Q/Q_{TFL}	MRGSHHHHHHGSIEGR VKE ITF**L/TFL**KNT APQM**L/TFL**RE **L/TFL**QETNAA **L/TFL**QDVRE**L/TFL** **L/TFL**RQQSK**L/TFL**	6.42/6.80
Q2/$Q2_{TFL}$	MRGSHHHHHHGSIEGR VKE **L/TFLL/TFL**F**L/TFL**KKT AEQM**L/TFL**EE **L/TFL**KETNKA **L/TFL**HDVRH**L/TFL** **L/TFL**ENQSK**L/TFL**	6.48/6.97

Table S2. Calculation results for TFL incorporation of $Q2_{TFL}$. Color-coded to calculation steps in **Equation 1**.

Incorporation	Sample 1	Sample 2	Sample 3	Average
% integrated area of incorporated peak	96.5%	92.5%	91.7%	**93.6 ± 0.6%**
% distance to total incorporated peak (6.97 kDa)	100%	99.8%	99.7%	**99.9 ± 0.2%**
Product of % incorporation	96.5%	92.4%	96.0%	**95.0 ± 2.3%**

Table S3. Calculation results for TFL incorporation of Q_{TFLl} corresponding to calculation steps in **Equation 1**.

Incorporation	Sample 1	Sample 2	Sample 3	Average
% integrated area of incorporated peak	100.0%	98.8%	100.0%	**99.5 ± 0.8%**
% distance to total incorporated peak (6.80 kDa)	100.0%	98.6%	100.0%	**99.6 ± 0.9%**
Product of % incorporation	100.0%	96.0%	100.0%	**98.7 ± 2.3%**

Table S4. MRE values calculated from CD measurements at 208 nm and 222 nm. Standard error is the result of three independent trials.

	Θ_{208} (deg•cm^2•dmol^{-1})	Θ_{222} (deg•cm^2•dmol^{-1})
**Q_{TFL}**	-500 ± 800	-4,300 ± 300
**$Q2_{TFL}$**	-100 ± 1,800	-15,000 ± 2,000

Table S5. ATR-FTIR compositional analysis from Q_{TFL}, $Q2_{TFL}$, and $Q2_{TFL}$-CCM. Summary of secondary structure content uses the average and standard deviation of the integrated area of deconvoluted peaks of three independent trials.

	% composition					
	**α-helix**	**β-sheet**	**Antiparallel β-sheet**	**3-10 helix**	**Unordered**	**Aggregated Strands**
**Q_{TFL}**	24.8 ± 6.8	30.6 ± 10.2	8.0 ± 8.8	18.6 ± 3.6	0.0 ± 0.0	17.4 ± 4.6
**$Q2_{TFL}$**	38.4 ± 14.0	28.7 ± 6.6	13.8 ± 5.5	12.4 ± 7.7	0.0 ± 0.0	6.8 ± 5.9
**$Q2_{TFL}$-CCM**	30.8 ± 6.9	21.7 ± 9.9	0.0 ± 0.0	23.5 ± 6.1	0.0 ± 0.0	9.8 ± 5.6

Table S6. SNR values calculated from $Q2_{TFL}$ and Q_{TFL} NMR spectra at various molar concentrations.

Concentration (mM)	$Q2_{TFL}$	Q_{TFL}
2.0	40.20	-
1.5	29.70	22.12
1.25	26.67	-
1	21.31	12.04
0.75	15.98	9.45
0.5	11.25	7.26
0.25	-	4.37

Table S7. SNR values calculated from $Q2_{TFL}$ at different TR and scan times using number of averages to maintain scan time at different TR.

TR (ms)	SNR (4 min.)	SNR (1 min.)
1000	19.94	7.79
800	20.9	-
500	22.6	11.06
400	-	-
300	25.85	11.87
200	27.87	11.57
100	32.77	14.35
80	29.84	-
50	28.78	11.3
40	-	-
30	28.54	-
26	-	11.7
